# Annexin A1 on the Surface of Early Apoptotic Cells Suppresses CD8^+^ T Cell Immunity

**DOI:** 10.1371/journal.pone.0062449

**Published:** 2013-04-30

**Authors:** Heiko Weyd, Lucie Abeler-Dörner, Björn Linke, Andrea Mahr, Veronika Jahndel, Sandra Pfrang, Martina Schnölzer, Christine S. Falk, Peter H. Krammer

**Affiliations:** 1 Tumor Immunology Program, German Cancer Research Center, Heidelberg, Germany; 2 Functional Proteome Analysis, German Cancer Research Center, Heidelberg, Germany; 3 Institute for Transplant Immunology, Hannover Medical School, Hanover, Germany; University of Michigan School of Medicine, United States of America

## Abstract

Prevention of an immune response against self-antigens derived from apoptotic cells is essential to preclude autoimmune and chronic inflammatory diseases. Here, we describe apoptosis induced externalization of endogenous cytosolic annexin 1 initiating an anti-inflammatory effector mechanism that suppresses the immune response against antigens of apoptotic cells. Cytosolic annexin 1 rapidly translocated to the apoptotic cell surface and inhibited dendritic cell (DC) activation induced by Toll like receptors (TLR). Annexin 1-inhibited DC showed strongly reduced secretion of pro-inflammatory cytokines (*e.g.* TNF and IL-12) and costimulatory surface molecules (*e.g.* CD40 and CD86), while anti-inflammatory mediators like PD-L1 remained unchanged. T cells stimulated by such DC lacked secretion of interferon-γ (IFN-γ) and TNF but retained IL-10 secretion. In mice, annexin 1 prevented the development of inflammatory DC and suppressed the cellular immune response against the model antigen ovalbumin (OVA) expressed in apoptotic cells. Furthermore, annexin 1 on apoptotic cells compromised OVA-specific tumor vaccination and impaired rejection of an OVA-expressing tumor. Thus, our results provide a molecular mechanism for the suppressive activity of apoptotic cells on the immune response towards apoptotic cell-derived self-antigens. This process may play an important role in prevention of autoimmune diseases and of the immune response against cancer.

## Introduction

Peripheral tolerance against self-antigens derived from apoptotic cells is essential for the organism as exemplified by deficiencies in the uptake of apoptotic cells which are associated with autoimmunity in mice and men [1], [2]. In the steady state, apoptotic cells continuously evolve in the course of tissue turnover and are rapidly removed by phagocytes, *e.g.* macrophages or DC [3]. Fast and efficient engulfment of apoptotic cells prevents accumulation of secondary necrotic cells and, thus, release of danger signals that can induce DC activation and autoimmunity [4]. Furthermore, apoptotic cells have been shown to actively suppress phagocytes such as macrophages, monocytes and DC *in vitro*, and engulfment of apoptotic cells is associated with inhibition of immune responses and the development of peripheral tolerance *in vivo* [5]–[7]. Under physiologic conditions, apoptotic cells are cleared within few hours and can be detected within DC in secondary lymphoid organs [8]. The phenotype of the phagocytosing DC determines further whether tolerance or immunity to self-antigens derived from apoptotic cells is initiated [9]. Although the default pathway following engulfment of apoptotic cells seems to be immune suppression and induction of tolerance, recent studies have identified a form of immunogenic cell death [10], emphasizing the importance of suppressive signals on apoptotic cells during physiological cell death in order to prevent immunogenicity [11]. Therefore, further analysis of the mechanisms by which apoptotic cells ensure self-tolerance is highly warranted.

Annexins are a family of evolutionary well conserved cytosolic proteins differentially expressed in various tissues [12]. All annexins bind to negatively charged phospholipids such as phosphatidylserine (PS) in a calcium-dependent manner; however, the physiological function of the twelve vertebrate annexins has not been fully elucidated yet. Annexin A1 has been described to regulate membrane dynamics and to mediate vesicle trafficking and membrane aggregation [13]. In addition to intracellular functions, annexin A1 has also been suggested to facilitate phagocytosis of apoptotic and secondary necrotic cells and to induce the recruitment of phagocytes [14]–[16]. *In vivo*, annexin A1 inhibits neutrophil and monocyte extravasation into inflamed tissue and, thus, ameliorates acute inflammation by binding and signaling through members of the formyl peptide receptor (FPR) family [17], [18]. Although anti-inflammatory effects of recombinant annexin A1 on monocytes and macrophages have been reported *in vitro* [16], [19], it has been unclear whether annexin A1 affects professional antigen presenting cells like DC and whether it also influences adaptive immune responses *in vivo*. Here, we show that annexin A1 translocates to the surface of apoptotic cells already at an early stage of apoptosis. Exposed annexin A1 then acts as an inhibitory effector molecule by which apoptotic cells suppress pro-inflammatory Toll-like receptor (TLR)- mediated signal transduction in DC. Annexin A1-inhibited DC display substantially reduced DNA-binding activity of the transcription factor NF-κB and diminished expression of pro-inflammatory genes such as cytokines (*e.g.* TNF and IL-12) and DC surface molecules (*e.g.* CD40 and CD86). However, annexin A1 did not inhibit immunosuppressive cytokines and molecules such as TGF-β and PD-L1. Thus, annexin A1 prevents the induction of inflammatory DC and facilitates the development of a tolerogenic DC phenotype. *In vivo*, annexin A1 prevents the induction of antigen-specific cytotoxic T cell responses and, consequently, impairs anti-tumor immunity and tumor rejection after vaccination against tumor antigens. Our results demonstrate that the early exposure of annexin A1 constitutes a molecular mechanism by which apoptotic cells gain their immunosuppressive phenotype to induce peripheral tolerance and prevent the development of autoimmune diseases.

## Results

### Annexin A1 is Exposed on the Surface of Early Apoptotic Cells

**Figure 1:**
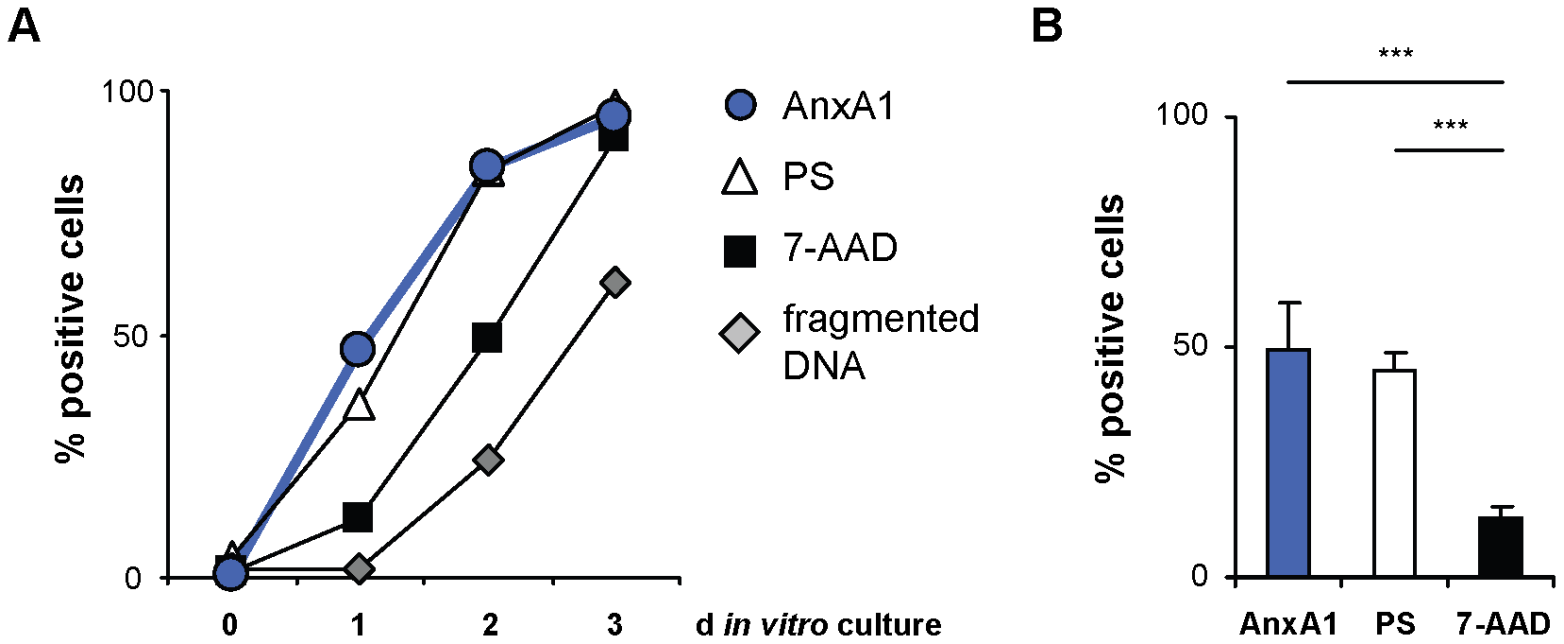
Annexin A1 is exposed on the surface of early apoptotic cells. (**A**) Primary human neutrophils were analyzed for characteristic features of apoptosis during continued aging *in vitro*. Externalization of PS, loss of membrane integrity and externalization of annexin A1 (AnxA1) was measured by flow cytometry using FITC-labeled annexin 5, 7-amino-actinomycin D (7-AAD) and FITC-labeled DAC5 antibody, respectively. Fragmented DNA = nuclei with fragmented DNA. (**B**) Primary human neutrophils of several donors after 1 d *in vitro* culture were analyzed as in (A). Error bars represent means +/− SD of several donors (n = 5). ***P<0.001.

Although many studies have demonstrated potent tolerogenic properties of apoptotic cells, only few suppressive molecules of apoptotic cells have been described so far [20]–[22]. To identify new anti-inflammatory molecules on the surface of apoptotic cells, we generated monoclonal antibodies directed against apoptotic cell-specific surface antigens. One of these antibodies, termed DAC5 (Detector of Apoptotic Cells No. 5), recognized apoptotic but not viable cells (Panel A in figure S1) and precipitated a protein of about 37 kDa, which was identified as annexin A1 by mass spectrometry (Panel B in figure S1). DAC5 detected annexin A1 on apoptotic primary human neutrophils and on apoptotic Jurkat T cells (Figures 1A, B and panels C, D in figure S1). A major fraction of these cells was not stained by the membrane impermeable DNA-dye 7-amino-actinomycin D (7-AAD) and, thus, had maintained membrane integrity (Figure 1B and panel D in figure S1). Kinetics showed that externalization of annexin A1 closely resembled the kinetics of the early apoptosis marker PS [23], preceding DNA fragmentation and loss of membrane integrity (Figures 1A, B and panel C in figure S1). Externalized annexin A1 could be detected after CD95 (Fas/APO-1) stimulation as well as after γ-irradiation or treatment with staurosporine (panel A in figure S2), demonstrating that exposure of annexin A1 was not dependent on the mode of apoptosis induction. Furthermore, all annexin A1-expressing cells tested, including primary human T cells and cell lines originating from various tissues, externalized the protein (Panel B in figure S2).

To confirm annexin A1 externalization during physiologic cell death we investigated primary human thymocytes, the majority of which die by apoptosis during negative selection at the CD4^+^/CD8^+^ double positive stage [24]. In accordance with the assumption that annexin A1 is present on early apoptotic thymocytes, DAC5 staining detected an annexin A1-positive fraction of thymocytes exclusively within the CD4^+^/CD8^+^ compartment (Panel C in figure S2). Moreover, the percentage of annexin A1-positive cells within total thymocytes correlated with the percentage of thymocytes that were positive for the early apoptosis marker PS (Panel C in figure S2). In conclusion, translocation of annexin A1 was detected at an early stage of apoptosis, when most cells had not lost membrane integrity, and occurred independently of the mode of apoptosis induction. Furthermore, upon apoptosis induction, we detected externalized annexin A1 on all annexin A1-expressing cell lines tested as well as on primary human thymocytes, T cells and neutrophils.

### Annexin A1 on the Surface of Apoptotic Cells Suppresses DC Activation

We hypothesized that externalized annexin A1 might mediate suppressive functions of apoptotic cells. Because the suppressive influence of apoptotic cells on DC has been well documented [25], [26], we analyzed the functional role of annexin A1 towards DC in further detail. First, we investigated the influence of apoptotic cells and annexin A1 on activation of DC and of a monocytic model cell line, U937, *in vitro*. As expected, apoptotic cells inhibited TLR-induced DC activation in a coculture assay (Figure S3). This effect was not seen when apoptotic cells were separated from DC through a transwell insert (Figure S4). Thus, suppression of DC by apoptotic cells was mediated mainly by cell bound factors rather than by released, soluble molecules.

**Figure 2:**
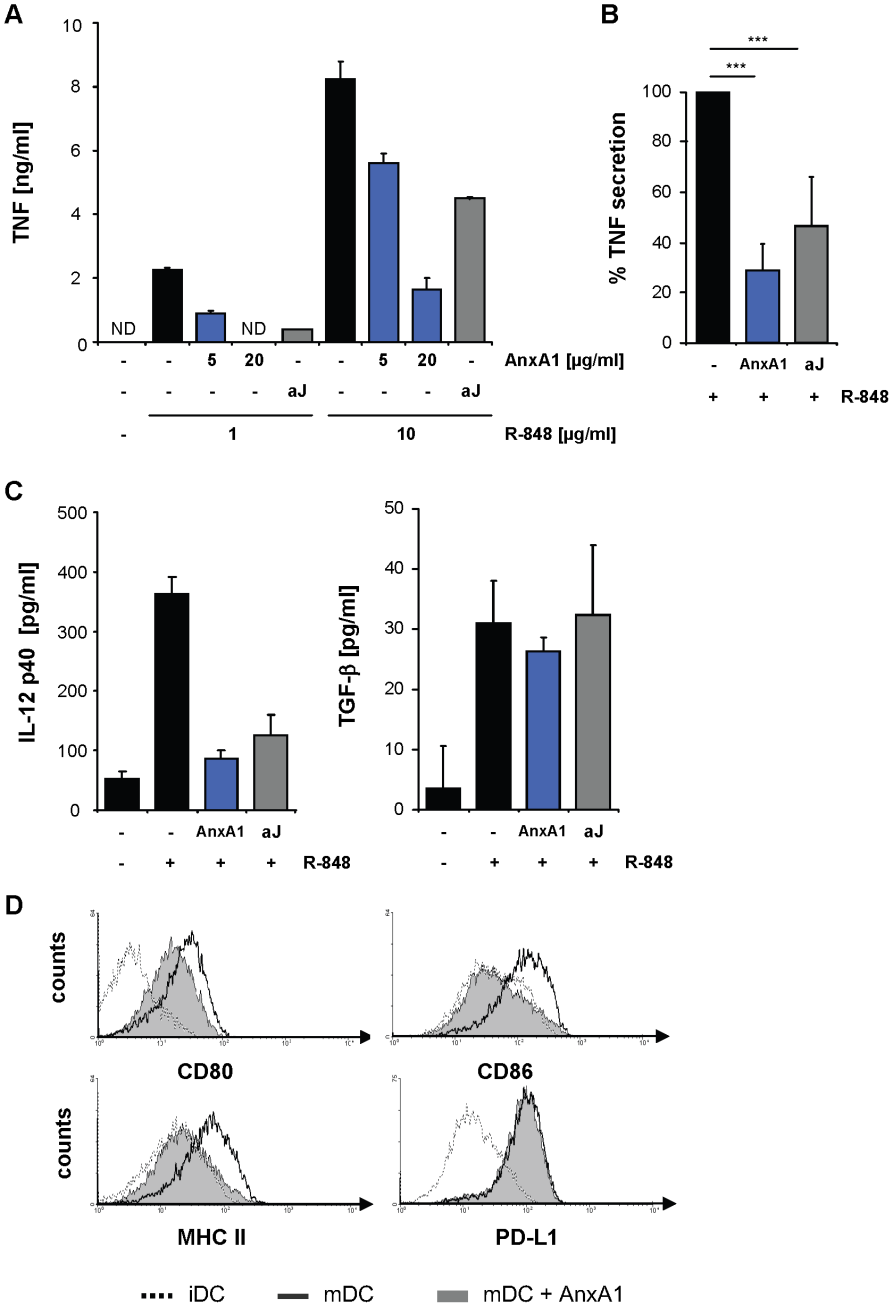
Annexin A1 suppresses DC activation. (**A**) Immature DC were incubated with the indicated concentrations of annexin A1 (AnxA1) or apoptotic Jurkat T cells (aJ), or left untreated. After stimulation with the indicated concentrations of R-848, TNF in culture supernatants was determined by ELISA. Error bars represent means +/− SD of duplicate cultures. ND = not detectable. (**B**) DC were treated as in (A). For several donors, the relative TNF secretion of treated DC was calculated relative to DC stimulated with R-848 only, which was set to 100%. Error bars represent means +/− SD (n = 7). ***P<0.001. (**C**) Secretion of IL-12 p40 and TGF-β from DC treated as in (A). Error bars represent means +/− SD of triplicate cultures. ND = not detectable. (**D**) Analysis of DC surface molecules. Untreated DC (iDC, dashed line) or DC stimulated by a cytokine cocktail after pre-incubation with annexin A1 (mDC+AnxA1, filled histograms) or without annexin A1 (mDC, bold lines) were analyzed by flow cytometry for the indicated molecules. Data are representative of at least 3 independent experiments. In experiments B to D, recombinant annexin A1 was used at a concentration of 5 or 10 µg/ml while R-848 was used at a concentration of 5 µg/ml.

Recapitulating this suppressive effect of apoptotic cells, treatment with recombinant annexin A1 suppressed TLR-induced inflammatory cytokine secretion of DC in a dose-dependent manner and inhibited upregulation of costimulatory surface molecules on DC matured by a cytokine cocktail [27] (Figures 2A–D). Remarkably, annexin A1 affected DC maturation differentially. Whereas secretion of TNF and IL-12 and expression of CD80, CD86 and MHC class II molecules were reduced almost to levels of immature DC, amounts of secreted TGF-β as well as expression of PD-L1 were not altered (Figures 2C, D). Thus, annexin A1 led to a DC phenotype characteristic of tolerogenic DC, featuring resistance to maturation as well as low expression of costimulatory surface molecules along with high expression of inhibitory ligands like PD-L1 [28].

Primary exposure of DC to danger signals like bacterial lipopolysaccharide (LPS) can render these cells refractory to subsequent stimulation, a phenomenon known as endotoxin tolerance [29]. Therefore, we used highly purified annexin A1 preparations and additionally included LPS-neutralizing polymyxin B in all experiments with recombinant protein. Furthermore, we verified the suppressive activity of recombinant annexin A1 in experiments with DC from TLR4^−/−^ mice. Similar to the effect of human annexin A1 on human DC, recombinant murine annexin A1 also induced a tolerogenic phenotype in murine DC in the absence of TLR4 (Figure S5). Heat-inactivated murine annexin A1 or a recombinant control protein (Serpin B8), purified in the same manner as recombinant annexin A1, were used as controls and did not exhibit suppressive activity (Figure S5). These results confirm a specific immunosuppressive effect of annexin A1 on human as well as on murine DC.

**Figure 3:**
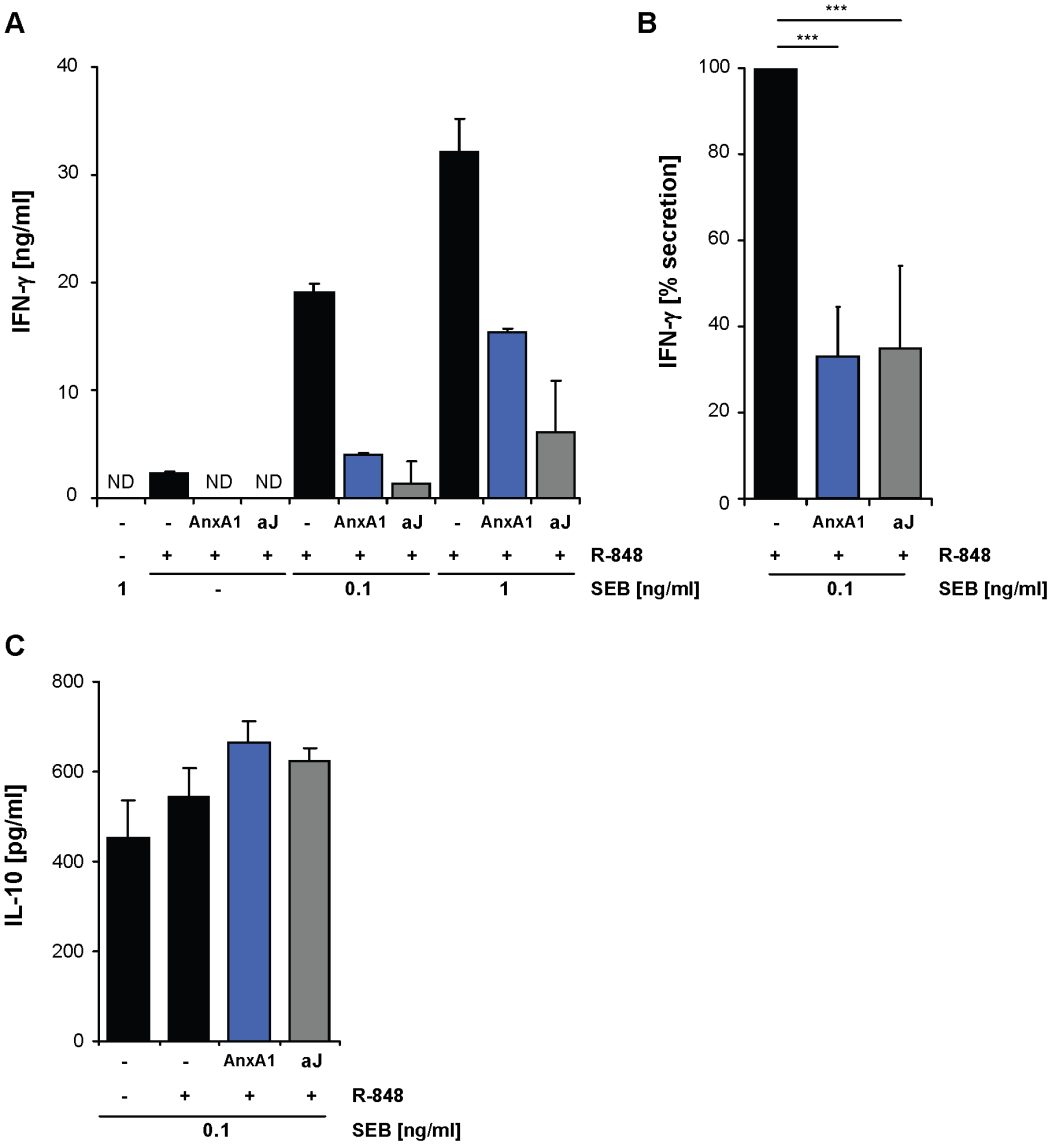
Annexin A1 influences DC-mediated T cell stimulation. (**A**) Immature DC were incubated with annexin A1 (AnxA1, 20 µg/ml), apoptotic Jurkat T cells (aJ), or left untreated. 2 days after stimulation by R-848, autologous T cells together with SEB in the indicated concentrations were added to the culture. The concentration of IFN-γ in culture supernatants was determined by ELISA after 6 days. Error bars represent means +/− SD of quadruplicate cultures. (**B**) For several donors, the relative IFN-γ secretion of cultures treated as in (A) is shown. IFN-γ-secretion in cultures with treated DC was calculated relative to cultures with DC stimulated by R-848 only, which was set to 100%. Error bars represent means +/− SD (n = 7). ***P<0.001. (**C**) Cocultures were set up as in (A) and concentrations of IL-10 in culture supernatants were determined by ELISA after 4 days. Error bars represent means +/− SD of triplicate cultures. Data are representative of at least 3 independent experiments. In all experiments, R-848 was used at a concentration of 5 or 10 µg/ml.

The phenotype of annexin A1-treated DC prompted us to investigate T cell responses initiated by such DC. We stimulated human CD4^+^ T cells with autologous TLR-stimulated DC along with the super-antigen SEB. In this coculture, T cell stimulation was dependent on addition of the superantigen SEB as well as on the presence of TLR-activated DC (Figure 3A). While immature DC failed to stimulate IFN-γ secretion by T cells, TLR-stimulated DC induced strong T cell activation, characterized by high levels of secreted IFN-γ (Figures 3A, B), which was T cell-derived as verified by intracellular FACS analysis (Figure S6). In marked contrast, T cells stimulated by annexin A1-treated, TLR-stimulated DC failed to secrete IFN-γ. Notably, secretion of IL-10 was induced in T cells by immature as well as by TLR-activated DC and remained unaffected by annexin A1 (Figure 3C). T cell stimulation by annexin A1-treated DC therefore reflects the differential effects of annexin A1 on DC phenotype, selectively suppressing pro-inflammatory cellular cues. The immunosuppressive effects of annexin A1 treatment could be attributed exclusively to DC, as T cells pre-treated directly with annexin A1 showed increased secretion of TNF and IFN-γ upon stimulation, as has been described before (Figure S7) [30].

**Figure 4:**
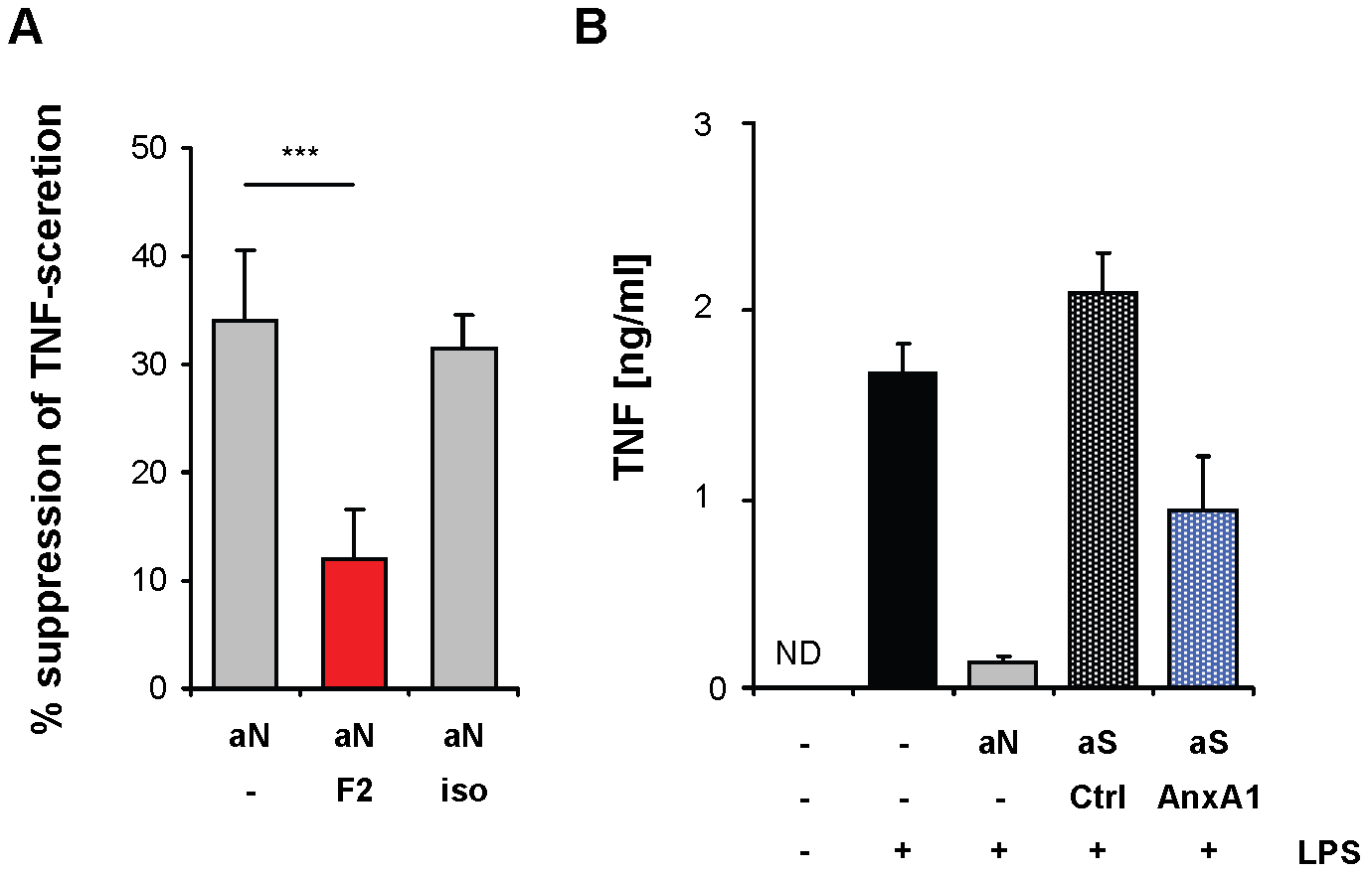
Apoptotic cells suppress DC activation *via* externalization of annexin A1. (**A**) Immature DC were incubated with apoptotic neutrophils (aN) together with anti-annexin A1 F(ab’)_2_-fragments (F2, 50 µg/ml) or isotype control F(ab’)_2_-fragments (iso, 50 µg/ml). After stimulation with CpG (0.05 µM) for 12 h, the concentration of TNF in culture supernatants was determined by ELISA. Depicted is the suppression of TNF secretion by apoptotic neutrophils in comparison to DC stimulated alone. Error bars represent means +/− SD of 3 independent experiments. ***P<0.001. (**B**) Immature DC were cocultured with apoptotic neutrophils (aN) or with apoptotic *Drosophila* Schneider cells (aS), transfected with annexin A1 (AnxA1) or a control plasmid (Ctrl). Subsequently, cultures were stimulated with LPS (1 ng/ml), and concentrations of TNF in culture supernatants were determined by ELISA. Error bars represent means +/− SD of duplicate cultures. Data are representative of 3 independent experiments.

Next, we investigated the contribution of annexin A1 to the inhibitory effect of apoptotic cells by blocking annexin A1 with rabbit anti-annexin A1–F(ab’)_2_-fragments. In a coculture assay using murine primary cells, apoptotic neutrophils suppressed TLR-induced TNF secretion by about 30%. This suppressive effect was markedly relieved when surface bound annexin A1 was blocked by addition of anti-annexin A1-F(ab’)_2_ fragments (Figure 4A), demonstrating that annexin A1 contributes substantially to the suppressive phenotype of apoptotic cells towards DC *in vitro*.

To complement these blocking experiments we also performed gain of function experiments. In order to examine the function of annexin A1 in the absence of other interfering apoptotic cell-derived signals we utilized apoptotic cells of an evolutionary distant species, reasoning that such cells are unlikely to provide intrinsic anti-inflammatory signals to mammalian DC. Indeed, control transfected apoptotic *Drosophila* Schneider cells (S2 cells) did not influence LPS-induced cytokine secretion of DC or U937 cells (Figure 4B and figure S8). This system enabled us to monitor the activity of human annexin A1 ectopically expressed in S2 cells. Like on apoptotic mammalian cells, transfected human annexin A1 was readily exposed on the surface of early apoptotic S2 cells (Figure S9 and figure S10). When added to human DC and U937, only the annexin A1-expressing apoptotic S2 cells exhibited a suppressive phenotype and inhibited LPS-induced inflammatory cytokine secretion of DC and U937 cells (Figure 4B and figure S8), confirming that expression of annexin A1 suffices to render apoptotic cells immunosuppressive.

### Annexin A1 Inhibits TLR-induced Signal Transduction Pathways in DC

**Figure 5:**
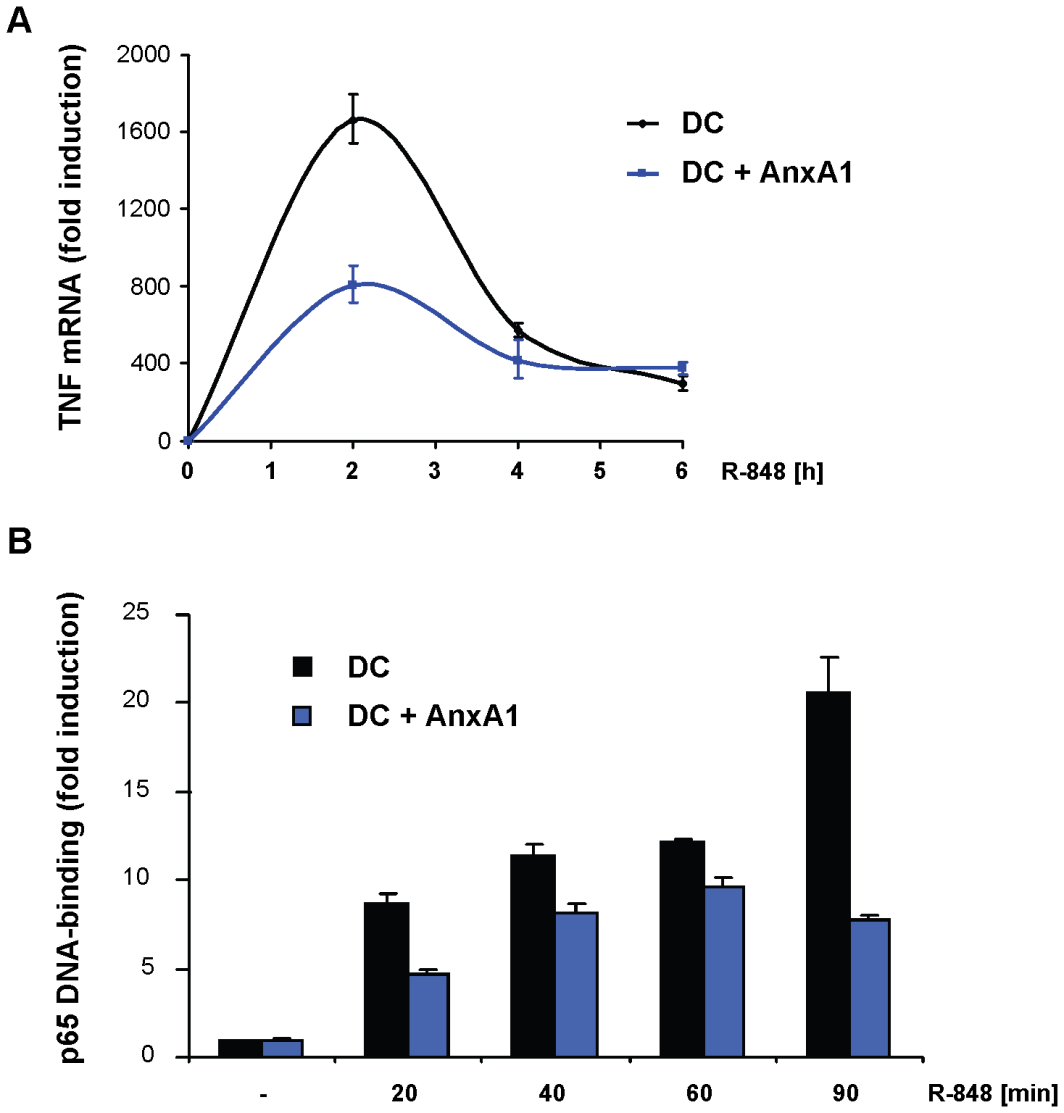
Annexin A1 inhibits TLR-induced signal transduction pathways. (**A**) Immature DC were incubated with annexin A1 (AnxA1, 20 µg/ml) or left untreated. Subsequently, DC were stimulated with R-848 (1 µg/ml) for the indicated time periods. TNF mRNA expression was determined by reverse transcription quantitative PCR normalized to GAPDH. Fold change was calculated relative to unstimulated DC (set to 1). Data are representative of at least 3 independent experiments. (**B**) Immature DC were treated as in (B). DNA binding of the NF-κB subunit p65 was analyzed in nuclear extracts of DC by ELISA. Error bars represent means +/− SD of duplicate measurements. Data are representative of 2 independent experiments.

Apoptotic cells arise continuously during tissue homeostasis. Therefore, apoptotic cell derived self-antigens are constantly presented on DC, and unwanted DC activation by endogenous or exogenous danger signals might lead to the development of autoimmunity [4], [31], [32]. Experiments presented in figure 4 show that annexin A1 suppressed DC activation induced by TLR4, which is engaged by endotoxins of gram-negative bacteria but also by endogenous danger signals [33]. In addition, annexin A1-mediated suppression was also detected after ligation of the endosomal receptors TLR7/8 and TLR9, triggered by viral ligands (Figure 2 and figure S5), confirming that annexin A1 can protect against unwanted DC activation induced by a broad spectrum of endogenous and exogenous danger signals. We next tested TLR-induced gene expression after annexin A1 treatment and detected significantly inhibited mRNA expression of TNF and further inflammatory cytokines (Figure 5A and figure S11). Gene expression following TLR stimulation is largely dependent on activation of the transcription factor NF-κB [34]. Therefore, we monitored the DNA-binding activity of the NF-κB subunit p65 in nuclear extracts of primary DC. In fact, pre-incubation with annexin A1 led to marked reduction of TLR-induced binding of p65 to DNA (Figure 5B). Thus, annexin A1 interferes with TLR signaling pathways upstream of canonical NF-κB activation in DC. To date, most functions of extracellular annexin A1 have been shown to be mediated through receptors of the FPR family [18]. However, the FPR-antagonist *N*-*t*-butoxycarbonyl-MLP (Boc-1) did not abrogate the suppressive effect of apoptotic cells or annexin A1 on DC (unpublished observation). Therefore, the tolerogenic receptor on DC that mediates the annexin A1 effect is likely different from receptors of the FPR family and remains subject to further investigations.

### Annexin A1 on Apoptotic Cells Inhibits Antigen-specific T Cell Responses *in vivo*

**Figure 6:**
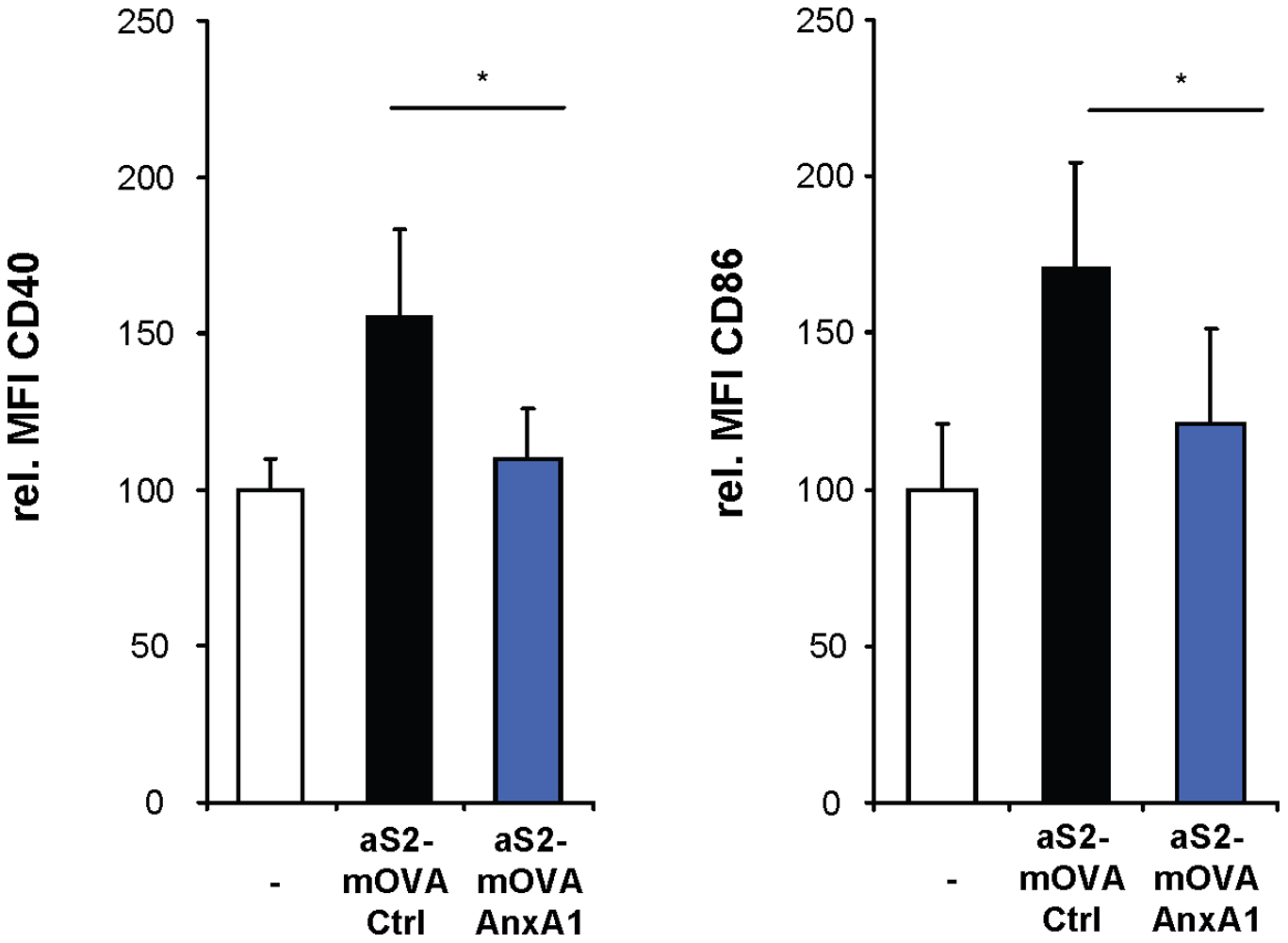
Annexin A1 inhibits DC activation *in vivo*. Apoptotic, mOVA-expressing *Drosophila* Schneider cells (aS2-mOVA) transfected with annexin A1 (AnxA1) or a control plasmid (Ctrl) were injected into mice. After 2 days, DC in lymph nodes of naïve (−) and S2-injected mice were analyzed for CD40 and CD86 expression by flow cytometry. Shown are quantifications of the MFI for CD40 and CD86 of total lymph node cells gated on CD11c^+^ and MHC class II^+^ cells *P<0.05 (n = 7). Data are representative of 3 independent experiments.

Immune suppression by apoptotic cells results in loss of antigen-specific CD8^+^ T cell effector function *in vivo* [9]. Because immunization with S2 cells has been used before to induce T cell-mediated immune responses [35], we took advantage of the fact that expression of annexin A1 conferred suppressive capacity to apoptotic S2 cells *in vitro*. First, we investigated the phenotype of DC after injection of mice with apoptotic S2 cells. Injected xenographic apoptotic S2 cells provided an inflammatory stimulus and led to maturation of lymph node DC within 2 days as detected by upregulation of the costimulatory molecules CD40 and CD86 (Figure 6 and figure S12). Notably, DC maturation was prevented when apoptotic S2 cells expressing murine annexin A1 were used for immunization (Figure 6), indicating a suppressive role for endogenous annexin A1 on DC *in vivo* which counteracted inflammation induced by injection of apoptotic S2 cells.

**Figure 7:**
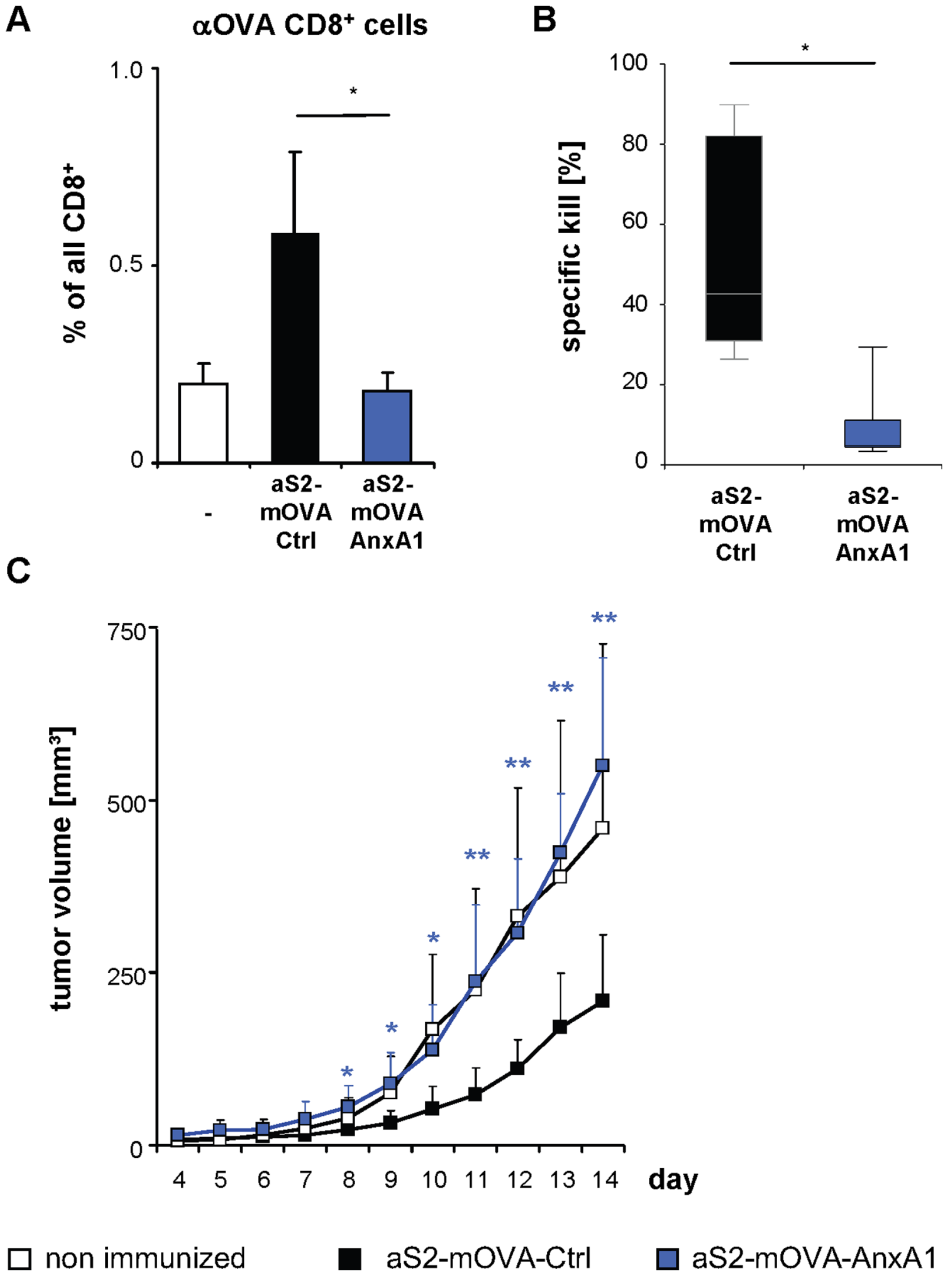
Annexin A1 suppresses immune responses *in vivo*. (**A**) Apoptotic, mOVA-expressing *Drosophila* Schneider cells (aS2-mOVA) transfected with annexin A1 (AnxA1) or a control plasmid (Ctrl) were injected into mice. After 8 days, percentages of OVA-specific CD8^+^ T cells relative to total CD8^+^ T cells in lymph nodes were analyzed by flow cytometry. *P<0.05 (n = 5). (**B**) Mice were immunized as in (A) and challenged with OVA/CpG after 10 days. CD8^+^ T cell function was analyzed by an *in vivo* cytolytic assay using OVA-peptide labeled target cells (n = 4–5), *P<0.05. (**C**) Mice were immunized as in (A). After 12 days, mice were injected with OVA-expressing B16 melanoma cells. Tumor growth was monitored for 4 to 14 days. *P<0.05, **P<0.01 for tumors of mice immunized with annexin A1-expressing apoptotic S2-mOVA cells compared to mice immunized with apoptotic S2-mOVA cells (n = 6). Data are representative of 3 (A) or 2 (B, C) independent experiments.

To analyze antigen-specific T cell responses in this system, we co-expressed a membrane-anchored form of ovalbumin (mOVA) in S2 cells (S2-mOVA) (Figure S13) and examined the course of the anti-OVA T cell response after immunization with apoptotic S2-mOVA cells. Injection of apoptotic S2-mOVA cells led to expansion of endogenous, OVA-specific CD8^+^ T cells (Figure 7A). Remarkably, injection of annexin A1-expressing apoptotic S2-mOVA cells prevented the expansion of OVA-specific CD8^+^ T cells (Figure 7A), in accordance with the observed reduction in DC maturation. To further investigate the effect of annexin A1 expression in apoptotic S2-mOVA cells on T cell function, we first immunized mice with apoptotic S2-mOVA cells and then monitored T cell effector function after *in vivo* restimulation with OVA in CpG. This challenge elicited a strong antigen-specific cellular immune response as demonstrated by *in vivo* cytotoxic activity (Figure 7B). This response was drastically reduced when mice had been injected with annexin A1-bearing apoptotic S2-mOVA cells (Figure 7B).

To investigate whether annexin A1-mediated inhibition of antigen specific immune responses against apoptotic cells extends to an anti-tumor response, we employed a model of preventive anti-tumor vaccination with apoptotic S2-mOVA cells and monitored the growth of OVA-transfected B16 melanoma cells (B16-OVA) [36]. As expected, growth of B16-OVA melanomas was significantly impaired following immunization with apoptotic S2-mOVA cells (Figure 7C). This anti-tumor effect was overcome, however, in mice immunized with annexin A1-expressing apoptotic S2-mOVA cells (Figure 7C). These results confirm that annexin A1 confers immunosuppressive properties to apoptotic cells *in vivo*, which prevents DC activation and impairs the development of a functional CD8^+^ T cell response. Consequently, annexin A1 on apoptotic cells compromises antigen-specific anti-tumor immunity and tumor rejection after vaccination against tumor antigens.

## Discussion

The prevention of immune responses against antigens derived from apoptotic cells is essential for the establishment and maintenance of peripheral self-tolerance [37]. Our results demonstrate that the exposure of annexin A1 confers a suppressive phenotype to apoptotic cells during early stages of apoptosis. Exposure of annexin A1 on early apoptotic cells, *i.e.* on cells that had not lost their cell membrane integrity, was detected on various cell lines and primary cells analyzed. Moreover, in addition to exposure of annexin A1 during apoptosis induced by artificial stimuli like irradiation or kinase inhibition by staurosporine, our data show that annexin A1 is also exposed during physiologic cell death, namely on aged neutrophils and on apoptotic thymocytes *ex vivo*. Finally, annexin A1 translocates to the surface of apoptotic cells early, at the time when apoptotic cell engulfment is thought to precede *in vivo* [38] and is, therefore, in place to provide signals to the phagocytosing DC.

The development of peripheral tolerance against self-antigens derived from apoptotic cells has been shown to critically depend on the phenotype of the self-antigen presenting DC [9]. We provide evidence that the exposure of annexin A1 on apoptotic cells supports a tolerogenic phenotype in primary DC, characterized by low expression of costimulatory surface molecules and inflammatory cytokines, reduced pro-inflammatory signal transduction and resistance to maturation [28]. Interestingly, the inhibitory ligand PD-L1, involved in cross tolerance towards self-antigens [39]–[41], was spared from annexin A1-mediated down regulation on DC. Thus, due to the suppressive activity of annexin A1, DC are likely to present antigens derived from apoptotic cells in a tolerogenic, non-danger context. We show that this tolerogenic DC phenotype indeed affected the stimulatory capacity towards autologous T cells *in vitro*, and prevented T cell derived IFN-γ secretion, while secretion of the immune regulatory cytokine IL-10 remained unchanged. These *in vitro* results indicate that annexin A1 prevents the development of a cytokine milieu known to contribute to Th1-mediated autoimmune disorders [42]. Furthermore, by using mOVA as a model antigen of apoptotic cells, we provide evidence that the annexin A1-mediated anti-inflammatory mechanism plays a role *in vivo* and prevents the induction of a CD8^+^ T cell mediated immune response. This was evidenced by reduced numbers and restrained function of antigen-specific CD8^+^ T cells upon immunization with apoptotic cells ectopically expressing annexin A1. This gain of suppressive activity upon ectopic annexin A1 expression in S2 cells together with our blocking experiments using anti-annexin A1 F(ab’)_2_-fragments on primary apoptotic cells demonstrates that annexin A1 largely contributes to the suppressive effect of cells undergoing physiological cell death.

The suppressive effect of apoptotic cells and annexin A1 was detectable under stimulated assay conditions only, using TLR-ligands *in vitro* and xenographic S2 cells *in vivo*. Still, the presence of immunosuppressive signals like annexin A1 and others [43] on apoptotic cells is likely beneficial under non inflammatory conditions as well, because some forms of cell death have been associated with immune activation [4], [44]. During physiologic tissue turnover a complex network comprising autophagy and mechanisms of programmed cell death ensures tissue homeostasis and rapid as well as immunological silent removal of excess or damaged cells [45], [46]. However, certain conditions favor a more immunogenic type of cell death. Following severe energy deprivation as in ischemia, during supraphysiologic conditions (like mechanical force, heat or toxins) or if cell death pathways are blocked cells will undergo necrotic cell death, characterized by organelle swelling and cellular membrane rupture [47], [48]. Thereby, intracellular molecules, so called DAMPS (danger-associated molecular patterns) like uric acid, HMGB1 and others are released and activate the immune system [49]. But even though DAMPS stimulate DC and activate the immune system [50], [51], tissue injuries only rarely initiate autoimmune diseases. Thus, Kono and Rock propose a delicate balance between inflammatory and anti-inflammatory cellular cues [49]. The need to ensure tolerance against antigens derived from apoptotic cells requires, therefore, anti-inflammatory signals like annexin A1 to be associated with apoptotic cells during normal tissue turnover. The persistent danger of immune activation by dying cells, if not counter balanced by immunosuppressive signals, is also highlighted by the an exceptional form of immunogenic cell death induced by certain chemotherapeutic agents like anthracyclins [11]. Next to exposure of calreticulin and release of ATP as well as HMGB1, immunogenic cell death is characterized by pre-apoptotic exposure of calreticulin and premature engulfment [10], [11]. At this stage, dying cells have not yet exposed PS on their surface [10] and, thus, no PS-binding signals like Gas6 or annexin A1 can counteract immunogenic signals.

Recently, exposure of annexin A1 has been detected on secondary necrotic cells [16], in contrast to the early exposure of annexin A1 on apoptotic cells described here. Because the amount of exposed annexin A1 correlates with its expression level (data not shown), differences in annexin A1 expression of the analyzed apoptotic cell population as well as in the affinity of the antibodies used may account for these divergent results.

The analysis of annexin A1-inhibited signaling revealed that annexin A1 treatment antagonizes TLR-induced signaling pathways irrespective of the particular TLR stimulated. Thereby, annexin A1 on apoptotic cells is able to protect DC presenting apoptotic cell-derived self-antigens from unwanted activation by a broad variety of endogenous and exogenous danger signals. Furthermore, inhibition by annexin A1 was evident at the level of NF-κB activity and led to inhibition of several pro-inflammory genes. However, not all NF-κB-dependent were genes were regulated by annexin A1, as exemplified by PD-L1 [52], suggesting a complex regulation of NF-κB-mediated signal transduction pathways by annexin A1. Annexin A1 has been shown to signal through receptors of the FPR family [18], but the FPR antagonist Boc-1 did not abrogate DC suppression by annexin A1 (data not shown). Therefore, our data point to the existence of a different, yet unknown tolerogenic annexin A1 receptor on DC.

Taken together, our results demonstrate that early externalization of annexin A1 contributes an essential tolerogenic property to apoptotic cells, conferring their ability to suppress DC activation and cellular immunity. This describes a new molecular mechanism, which facilitates the development of peripheral tolerance towards antigens derived from apoptotic cells. Preclinical studies have shown that tolerogenic DC can be utilized to diminish transplant rejection and improve autoimmune diseases [53]. Along these lines, treatment with annexin A1 in conjunction with defined auto-antigens or infusion with annexin A1-treated allogenic DC might prove therapeutically beneficial, exploiting the physiologic mechanism of annexin A1-mediated immune suppression that we describe. Vice versa, cancer therapies like irradiation and cytotoxic drugs aim at inducing apoptosis in tumor cells. Here, the anti-tumor effect could be maximized if CD8^+^ T cell immunity could be directed against tumor antigens. Blocking anti-inflammatory mediators like exposed annexin A1 that arise due to treatment induced tumor cell apoptosis could tip the balance from an anti- to a pro-inflammatory tumor environment and, thus, facilitate tumor antigen recognition and tumor rejection. The notion that certain tumor entities, when exposed to the immune system, might exploit the immunosuppressive effect of annexin A1 is supported by the analysis of annexin A1 expression in some immunogenic tumor entities like melanoma [54]. Therefore, interference with the suppressive function of annexin A1 is likely to be a promising approach for novel anti-cancer therapies in the future.

## Materials and Methods

### Mice

TLR4^−/−^ deficient mice were kindly provided by S. Akira, S. Uemattsu and L. Gissmann. C57BL/6 mice were purchased from the Jackson Laboratory. All mice were maintained in specific-pathogen-free facilities.

### Antibodies and Reagents

Human cell surface molecules were stained with antibodies against PD-L1 (eBioscience, Frankfurt, Germany), CD25, CD62L, CD69, CD80, CD86, HLA-DR, HLA-A, B, C or the appropriate isotype control antibodies (BD Pharmingen, Heidelberg, Germany). Murine cell surface molecules were stained with antibodies against I-A/I-E, CD11c, CD40 and CD86 and CD90.1 or the appropriate isotype control antibodies (Biolegend, Fell, Germany). PS exposure and cellular membrane integrity were analyzed with annexin A5 (Immunotools, Friesoythe, Germany) and 7-amino-actinomycin D (7-AAD, Sigma, Munich, Germany), respectively. Cytokine concentrations in supernatants were determined by ELISA for human and murine TNF, human IFN-γ (BD Pharmingen, Heidelberg, Germany), human IL-10 (Immunotools, Friesoythe, Germany) and TGF-β (R&D Systems, Wiesbaden, Germany), according to the manufacturer’s instructions. Recombinant proteins were expressed in the *Escherichia coli* BL21(DE3)pLysS strain (Promega, Mannheim, Germany) was from the pET41a vector (Novagen/Merck, Darmstadt, Germany). PCR products of annexin A1 or serpin B8 were cloned into pET41a harboring a C-terminal FLAG-tag, a PreScission protease cleavage site and a Protein A-tag. Removal of LPS was achieved by washing with TBS containing 0.1% Triton X-114 (Sigma, Munich, Germany). LPS content in all annexin A1 preparations was determined to be below 5 EU/mg using the Limulus Amoebocyte Lysate Assay (Lonza, Cologne, Germany) according to the manufacturers’ instructions.

### Cells and Cell Lines

Human immature DC (iDC) and human CD4^+^ T cells were prepared from human peripheral blood mononuclear cells of healthy donors after informed consent. For preparation of immature DC, CD14^+^ monocytes were purified using anti-CD14 MACS-beads (Miltenyi, Bergisch-Gladbach, Germany) according to the manufacturer’s instructions and differentiated in DC differentiation medium (RPMI 1640 medium containing 1,000 U/ml human GM-CSF (Sargramostim, Bayer, Leverkusen, Germany), 500 U/ml human IL-4 (Immunotools, Friesoythe, Germany) and 1% human AB Serum (Sigma)) for 6 days. Fresh cytokines were supplemented on d 3. Human CD4^+^ T cells were prepared using a CD4^+^ T cell isolation kit II (Miltenyi, Bergisch-Gladbach, Germany) according to the manufacturer’s instructions. Human neutrophils were prepared by density gradient centrifugation using. Murine DC were prepared from bone marrow and cultured in RPMI 1640 medium/10% fetal bovine serum (FBS, Gibco/Life Technologies, Darmstadt, Germany), 2 mM glutamine and 10% supernatant of the GM-CSF-producing cell line GM-PC for 7 d. Murine GR1^+^ neutrophils were purified using anti-GR1 MACS-beads (Miltenyi, Bergisch-Gladbach, Germany) according to the manufacturer’s instructions. Human Jurkat T cells and the human T-ALL cell line CEM were cultured in RPMI 1640 medium supplemented with 10% FBS. *Drosophila* Schneider cells (DSMZ, Braunschweig, Germany) were cultured in Schneider’s S2 Medium (Sigma, Munich, Germany) supplemented with 10% FBS.

### Preparation of Apoptotic Cells

For induction of apoptosis, Jurkat T cells were irradiated with 75 mJ/cm^2^ UV-C in a Stratalinker 1800 (Stratagene/Agilent Technologies, Karlsruhe, Germany) and used after 2–3 h of incubation. *Drosophila* S2 cells were irradiated with 300 mJ/cm^2^ and used after overnight incubation. Apoptotic human neutrophils were obtained by continuous *in vitro* culture for 1–3 days.

### Generation of Antibodies against Apoptotic Cells

The DAC5 Hybridoma was generated from C57BL/6 mice immunized i.p. with 2×10^7^ apoptotic Jurkat T cells after incubation with staurosporine (1 µM, Sigma, Munich, Germany) for 0.5 h. For blocking experiments, rabbits were immunized with full length recombinant murine annexin A1. Total IgG was purified from rabbit immune serum by protein A-sepharose (Sigma, Munich, Germany), and anti-annexin A1 and isotype control F(ab’)_2_-fragments were prepared from total IgG by digestion with immobilized trypsin (Pierce/Thermo Scientific, Bonn, Germany) according to the manufacturer’s instructions. F(ab’)_2_-fragments were used at a final concentration of 50 µg/ml.

### Analysis of Human Thymic Tissue

Normal human thymocytes were isolated by passing cells through a strainer into ice cold PBS/10% FBS and stained with fluorophore-conjugated antibodies.

### Cocultures of DC, annexin A1, Apoptotic Cells and T Cells

All assays were performed with highly purified annexin A1 (LPS <5 EU/mg) and LPS-neutralizing polymyxin B (10 µg/ml, Sigma, Munich, Germany). 1×10^5^ iDC were incubated with recombinant annexin A1 (5–20 µg/ml) overnight or with apoptotic cells (5×10^5^ apoptotic human cells and 1×10^6^ apoptotic S2 cells) for 4–6 h. Subsequently, cultures were stimulated with LPS (1–1000 ng/ml), with R-848 (1–10 µg/ml, all Invivogen, Toulouse, France) or a cytokine cocktail [55]. Cytokine concentrations were analyzed 12–15 h after TLR stimulation while DC were analyzed for surface molecules 48 h after stimulation. In some experiments, autologous CD4^+^ T cells together with 0.1 ng/ml SEB were added to the wells and analyzed for T cell cytokines after 4–6 days.

### *In vivo* Experiments

For immunization, 1×10^7^ apoptotic S2 cells were injected into C57BL/6 wt mice i.v. After 2 days, single cell suspensions of explanted mesenteric lymph nodes were analyzed. Endogenous anti-OVA-specific CD8^+^ T cells were analyzed 6–8 days after immunization, using phycoerythrin-labeled Kb/SIINFEKL pentamers according the manufacturer’s instructions (ProImmune, Oxford, UK). 10 days after immunization, mice were challenged with 50 µg OVA and 30 µg CpG s.c. (both Invivogen, Toulouse, France). On day 7 after challenge, *in vivo* cytolytic activity was analyzed in splenocytes as previously described [56]. For tumor experiments, immunized mice were injected s.c. with 2.5×10^5^ B16 melanoma cells (clone MO4 [36]). Tumor growth was monitored by caliper for 2 weeks.

### RNA Preparation and Quantitative RT-PCR

RNA was isolated from 0.5×10^6^ DC after 2–3 h of stimulation with R-848 (1 µg/ml) using the RNAqueous Micro Kit (Ambion/Life Technologies, Darmstadt, Germany). RNA was quantified by detection of incorporated SYBR® Green using the ABI Prism 7500 sequence detector system (Applied Biosystems/Life Technologies, Darmstadt, Germany). The relative expression level was determined by normalization to GAPDH or HPRT1, and results are presented as fold induction compared to control sample, which were set to 1. Following primer sequences were used: GAPDH: 5′- TCG CCC CAC TTG ATT TTG (forward) and 5′- GAG GGA TCT CGC TCC TGG AAG A (reverse). HPRT1∶5′- TGA CAC TGG CAA AAC AAT GCA (forward) and 5′- GGT CCT TTT CAC CAG CAA GCT (reverse). TNF: 5′- GCC GCA TCG CCG TCT CCT AC (forward) and 5′- AGC GCT GAG TCG GTC ACC CT (reverse). IL1A: 5′- AGA TGG CCA AAG TTC CAG ACA (forward) and 5′- TGA TCC ATG CAG CCT TCA TG (reverse). IL6∶5′- CTC CAC AAG CGC CTT CGG TCC (forward) and 5′- GTG GCT GTC TGT GTG GGG CG (reverse). IL12B: 5′- GCA GAG GCT CTT CTG ACC CCC A (forward) and 5′- GCA GGC ACT GTC CTC CTG GC (reverse). IL23A: 5′- GTT CCC CAT ATC CAG TGT GG (forward) and 5′- AGT AGG GAG GCA TGA AGC TG (reverse).

### Determination of NF-κB DNA-binding

Immature DC were seeded at a density of 5×10^5^ cells per well in 12 well plates and incubated overnight with 5 µg/ml annexin A1. For analysis of NF-κB activity, nuclear extracts were prepared acccording to the manufacturer’s instruction (Active Motif, La Hulpe, Belgium). Protein concentration was determined and equal amounts of protein were loaded onto an NF-κB transcription factor ELISA (p65, Active Motif, La Hulpe, Belgium).

### Statistical Analysis

Comparisons between two groups were performed by 2-sample Student’s t-test, 2-tailed. The analysis of results depicted in figures 4B, 6B and 8C were performed using 1-sample Student’s t-test, 2-tailed. Data were analyzed using SigmaPlot (Vers. 12.10). p-values of less than 0.05 were considered statistically significant.

### Ethics Statement

All blood donations were performed after written informed consent to participate in this study and were approved by the Ethics Committee (EC) of the Medical Faculty Heidelberg (IRB00003441; approval S-403 2009). All animal studies were approved by the veterinary authorities, state of Baden-Wurttemberg (approvals G-96 06 and G-138 11). Normal human thymic tissue was obtained after written informed parental consent following the guidelines of the Institutional Review Board (Ethics Committee of the Tuebingen University Hospital, IRB00001526) from 2-day to 14-year old children undergoing corrective cardiac surgery at the Tuebingen University Hospital (approval AZ 302/2003V). As stated in the written consent and approved by the IRB00001526, thymic tissue that had to be removed inevitably in the course of medical indicated surgery was donated for general scientific purposes specifically including biochemical analyses as performed in the present study. No research was conducted outside of Germany.

## Supporting Information

Figure S1 **The antibody DAC5 binds to annexin A1 on early apoptotic cells.** (**A**) Apoptotic CEM cells treated for 4 h with staurosporine (blue histogram) or viable CEM cells (bold line) were incubated with the antibody DAC5 or an IgG2a isotype control antibody (isotype control, dashed line), followed by incubation with FITC-labeled anti-mouse IgG antibodies. (**B**) Immunoprecipitation with the antibody DAC5 (DAC5), an IgG2a isotype control antibody (iso), or without antibody (−) from lysates of apoptotic CEM cells. Proteins were resolved by SDS PAGE and detected by silver stain. The protein band labeled “AnxA1” was cut from the gel and identified as annexin A1 by mass spectrometry. *hc/lc = antibody heavy/light chain. (**C**) Characteristic features of apoptosis were analyzed in staurosporine-treated Jurkat T cells (Sts, 1 µM) after the indicated time periods by flow cytometry. Externalization of PS and loss of membrane integrity was assessed by staining with FITC-labeled annexin A5 and 7-Amino-actinomycin D (7-AAD), respectively. Exposure of annexin A1 (AnxA1) was analyzed using FITC-labeled DAC5 antibody or an isotype control antibody (iso). (**D**) Representative individual dot plots of apoptotic Jurkat T cells exposed to 75 mJ/cm^2^ UV-C irradiation and subsequently incubated for 2 hours. Exposure of PS and annexin A1 (AnxA1) was analyzed by staining with FITC-labeled annexin A5 and FITC-labeled DAC5 antibody, respectively. Percentages indicate annexin A1 (AnxA1)-positive (left panel) and PS-positive (right panel) apoptotic cells subdivided into early apoptotic cells with intact cell membrane (red, 7-AAD-negative) and late apoptotic cells (gray, 7-AAD positive). Data are representative of more than 3 independent experiments.
(TIFF)

Figure S2 **Annexin A1 is externalized after different stimuli and on apoptotic cells of different origin.** (**A**) Jurkat T cells were rendered apoptotic by irradiation with 150 Gy (Irrad), by incubation with staurosporine (Sts, 1 µM) or leucine zipper CD95 ligand (CD95L, 100 ng/ml) for 8 h. Externalization of PS, loss of membrane integrity and externalization of annexin A1 (AnxA1) was measured by flow cytometry using FITC-labeled annexin 5 and FITC-labeled DAC5 antibody. (**B**) The indicated cell types were rendered apoptotic by following treatments: activated primary human T cells and the cervix carcinoma cell line HeLa were incubated with staurosporine (1 µM), the hepatoma cell line HepG2 and the melanoma cell line A375 were irradiated with 150 Gy and 300 mJ/cm^2^ UV-C, respectively. Externalization of annexin A1 (AnxA1) was determined by flow cytometry using FITC-labeled DAC5 antibody (filled histograms), while membrane integrity was monitored by staining with 7-AAD. DAC5 staining on 7-AAD-negative cells is shown. The dashed line represents unstained cells. (**C**) Total human thymocytes were analyzed by flow cytometry for expression of CD4 and CD8. Staining with 7-AAD was used to exclude late apoptotic and necrotic cells. Externalized annexin A1 on 7-AAD negative cells was detected by FITC-labeled DAC5 antibody. Total thymocytes (left dot plot) and annexin A1-positive thymocytes (AnxA1^+^, right dot plot) are shown with respect to their CD4/CD8 expression. In the histograms on the left the percentages of PS-positive (PS) and annexin A1-positive (AnxA1) cells of total 7-AAD-negative thymocytes are indicated. Data are representative of at least 3 independent experiments.
(TIFF)

Figure S3 **Apoptotic cells suppress TLR induced DC-activation.** (**A**) Human DC were incubated with apoptotic neutrophils (aN) or apoptotic Jurkat T cells (aJ) for 4 h, or left untreated. After stimulation with the indicated concentrations of LPS for 12 hsecreted cytokines in culture supernatants were quantified by multiplex analysis. (**B**) For analysis of DC surface molecules, DC were pre-incubated with apoptotic Jurkat T cells as in (A) and subsequently stimulated by a cytokine cocktail for 2 days (mDC) or left untreated (iDC). iDC = untreated DC, dashed line; mDC = DC stimulated alone, bold line; mDC+aJ = DC stimulated after pre-incubation with apoptotic Jurkat T cells, filled histogram. (**C**) PMA-differentiated U937 cells were incubated with apoptotic neutrophils (aN) or apoptotic Jurkat T cells (aJ) for 4 h or left untreated, and subsequently stimulated with LPS (10 ng/ml) for 12 h. TNF concentrations in culture supernatants were determined by ELISA. Error bars represent means +/− SD of triplicate cultures. Data are representative of more than 3 independent experiments.
(TIFF)

Figure S4 **Suppression of DC by apoptotic cells is cell contact dependent.** (**A, B**) Immature DC (A) or differentiated U937 cells (B) were incubated with apoptotic neutrophils (aN) or apoptotic Jurkat T cells (aJ) directly or in a transwell insert (aNtw; aJtw; 1 µm pore size) for 4 h. After stimulation with LPS (10 ng/ml) for 12–16 h, the concentration of TNF in culture supernatants was determined by ELISA. ND = not detectable. Error bars represent means +/− SD of duplicate wells. Data are representative of 3 independent experiments.
(TIFF)

Figure S5 **Annexin A1 suppresses TLR4^−/−^ DC.** Immature DC from TLR4^−/−^ mice were incubated for 8 h with recombinant murine annexin A1 (AnxA1), heat inactivated annexin A1 (h.i. AnxA1), or recombinant serpin B8 as a control protein at the indicated concentrations. Subsequently, DC were stimulated with CpG (0.05 µM) for 12 h. TNF concentrations in culture supernatants were determined by ELISA. Error bars represent means +/− SD of duplicate cultures. Data are representative of more than 3 independent experiments. ND = not detectable.
(TIFF)

Figure S6 **Intracellular T cell cytokines after stimulation by annexin A1-treated DC.** Immature human DC were incubated overnight with annexin A1 (20 µg/ml, AnxA1), apoptotic Jurkat T cells (aJ), or left untreated. 2 days after maturation with R-848 (2.5 µg/ml), autologous CD4^+^ T cells together with SEB (0.1 ng/ml) were added to the culture wells. Intracellular T cell cytokines were analyzed by flow cytometry after 6 days of coculture with DC. Percentages of CD69^+^ and cytokine expressing T cells are indicated. Data are representative of 3 independent experiments.
(TIFF)

Figure S7 **Enhanced Th1 cytokine secretion in annexin A1-treated T cells.** CD4^+^ Tcells purified by magnetic beads (>95% purity) were incubated overnight with annexin A1 (20 µg/ml, AnxA1) followed by stimulation with agonistic antibodies against CD3 and CD28 together with IL-2 (TCR/IL-2; 1 µg/ml, 0.5 µg/ml and 25 U/ml, respectively) for 6 days. Subsequently, concentrations of cytokines in culture supernatants were determined by ELISA. Error bars represent means +/− SD of duplicate cultures. Data are representative of 3 independent experiments.
(TIFF)

Figure S8 **Apoptotic cells suppress activation of U937 cells** ***via*** **annexin A1.** PMA-differentiated U937 cells were cocultured with apoptotic neutrophils (aN) or apoptotic *Drosophila* Schneider cells (aS) transfected with annexin A1 (AnxA1) or with a control plasmid (Ctrl). Subsequently, cultures were stimulated with LPS (5 ng/ml) for 12 h. TNF concentrations in culture supernatants were determined by ELISA. Error bars represent means +/− SD of duplicate cultures. Data are representative of 3 independent experiments.
(TIFF)

Figure S9 **Transfected annexin A1 is externalized on apoptotic** ***Drosophila*** **Schneider cells.** (**A**) *Drosophila* Schneider cells (S2) were transfected with human annexin A1 (AnxA1) or an empty control vector (Ctrl). Lysates of transfected *Drosophila* Schneider cells and Jurkat T cells (J) were analyzed for expression of annexin A1 (AnxA1) and tubulin (Tub). (**B**) 72 h after transfection, UV-C-irradiated (300 mJ/cm^2^, 12 h), apoptotic *Drosophila* Schneider cells transfected with annexin A1 (S2-AnxA1, filled histogram) or a control plasmid (S2-Ctrl, bold line) were stained with FITC-labeled DAC5 antibody. (**C**) Apoptotic *Drosophila* Schneider cells transfected with annexin A1 as in (B) were tested for annexin A1 externalization using FITC-labeled DAC5 antibody, while membrane integrity was monitored by staining with 7-AAD. Percentages indicate annexin A1 (AnxA1)-positive cells subdivided into early apoptotic cells with intact cell membrane (7-AAD-negative) and late apoptotic cells (7-AAD positive). Data are representative of more than 3 independent experiments.
(TIFF)

Figure S10 **Kinetics of S2 cell apoptosis.** (**A–C**) *Drosophila* S2 cells expressing membrane-anchored ovalbumin and murine annexin A1 (S2-mOVA-AnxA1) or a control plasmid (S2-mOVA-Ctrl) were irradiatied with UV-C light (300 mJ) and analyzed after indicated time periods by flow cytometry. Kinetics (A) and representative histograms of PS-exposure (B) are shown. Externalization of PS and loss of membrane integrity were assessed by staining with FITC-labeled annexin A5 and 7-Amino-actinomycin D (7-AAD), respectively. Nuclei with fragmented DNA were detected as described by Nicoletti *et al*. In parallel, aliquots of cells were resuspended in PBS/EDTA (5 mM). Murine annexin A1 (AnxA1) in lysates and EDTA-washes (cell surface) of S2-mOVA-AnxA1 cells was detected on Western blot using a rat monoclonal antibody generated in our lab (C). Error bars represent means +/− SD of 3 experiments.
(TIFF)

Figure S11 **Annexin A1 suppresses gene transcription of NF-κB-dependent cytokines.** Human DC were incubated with annexin A1 (10 µg/ml) or left untreated. Subsequently, DC were stimulated with R-848 (1 µg/ml) for 2–3 h, and mRNA expression was analyzed by reverse transcription quantitative PCR. Shown are mRNA expression data of annexin A1-treated, TLR-stimulated samples relative to TLR-stimulated samples only (reference). Error bars represent means +/− s.e.m. *P<0.05; **P<0.001 (n = 5–12 donors).
(TIFF)

Figure S12 **Annexin A1 inhibits DC activation** ***in vivo*** **.** Apoptotic, mOVA-expressing *Drosophila* Schneider cells (aS2-mOVA) transfected with annexin A1 (AnxA1) or a control plasmid (Ctrl) were injected into mice. After 2 days, DC in lymph nodes of S2-injected mice were analyzed for CD40 and CD86 expression by flow cytometry. Shown are representative histograms of lymph node cells of mice injected with the indicated apoptotic S2 cells and gated on CD11c^+^ and MHC class II^+^ cells. Data are representative of 3 independent experiments.
(TIFF)

Figure S13 **Annexin A1 and mOVA expression in S2 cells injected into mice.** Lysates of S2 cells (5 million cells/lane) stably transfected with mOVA (S2-mOVA) and murine annexin A1 (AnxA1) or a control vector (Ctrl) were analyzed on Westernblot using antibodies against OVA, murine annexin A1 and tubulin (Tub), respectively. For comparison, lysates of untransfected S2 cells (S2), of the murine macrophage cell line J774 (1 million cells/lane) and recombinant OVA (rOVA, 2.5 ng/lane) were loaded in parallel.
(TIFF)
