# Supplementary figures and images for: Annexin A1 on the Surface of Early Apoptotic Cells Suppresses CD8+ T Cell Immunity
Source: PLoS One. 2013 Apr 30;8(4):e62449. doi: 10.1371/journal.pone.0062449 (PMC3640057; doi:10.1371/journal.pone.0062449)

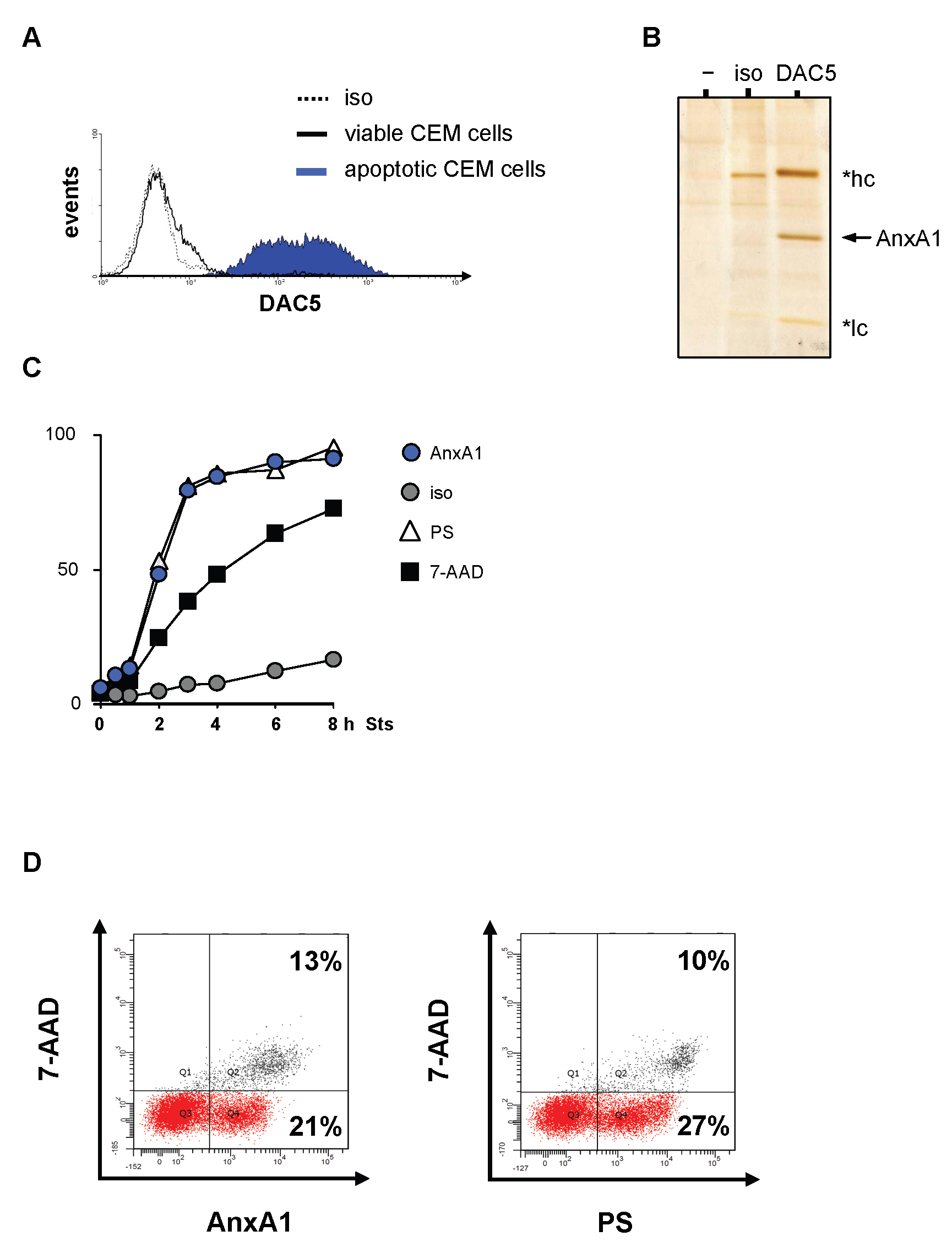

Supplement: Figure S1 — The antibody DAC5 binds to annexin A1 on early apoptotic cells. (A) Apoptotic CEM cells treated for 4 h with staurosporine (blue histogram) or viable CEM cells (bold line) were incubated with the antibody DAC5 or an IgG2a isotype control antibody (isotype control, dashed line), followed by incubation with FITC-labeled anti-mouse IgG antibodies. (B) Immunoprecipitation with the antibody DAC5 (DAC5), an IgG2a isotype control antibody (iso), or without antibody (−) from lysates of apoptotic CEM cells. Proteins were resolved by SDS PAGE and detected by silver stain. The protein band labeled “AnxA1” was cut from the gel and identified as annexin A1 by mass spectrometry. *hc/lc = antibody heavy/light chain. (C) Characteristic features of apoptosis were analyzed in staurosporine-treated Jurkat T cells (Sts, 1 µM) after the indicated time periods by flow cytometry. Externalization of PS and loss of membrane integrity was assessed by staining with FITC-labeled annexin A5 and 7-Amino-actinomycin D (7-AAD), respectively. Exposure of annexin A1 (AnxA1) was analyzed using FITC-labeled DAC5 antibody or an isotype control antibody (iso). (D) Representative individual dot plots of apoptotic Jurkat T cells exposed to 75 mJ/cm2 UV-C irradiation and subsequently incubated for 2 hours. Exposure of PS and annexin A1 (AnxA1) was analyzed by staining with FITC-labeled annexin A5 and FITC-labeled DAC5 antibody, respectively. Percentages indicate annexin A1 (AnxA1)-positive (left panel) and PS-positive (right panel) apoptotic cells subdivided into early apoptotic cells with intact cell membrane (red, 7-AAD-negative) and late apoptotic cells (gray, 7-AAD positive). Data are representative of more than 3 independent experiments. (TIFF) [file pone.0062449.s001.tiff]

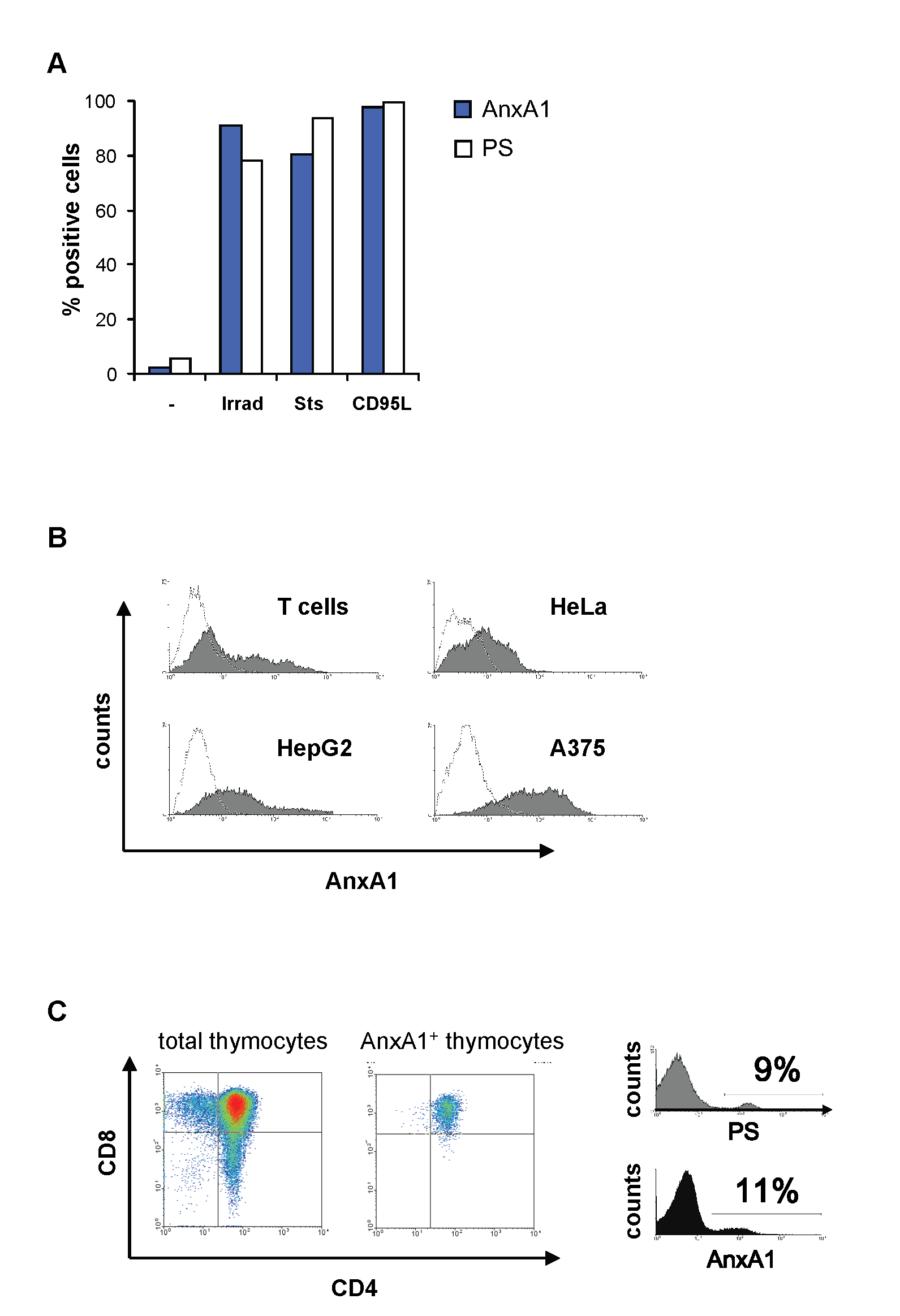

Supplement: Figure S2 — Annexin A1 is externalized after different stimuli and on apoptotic cells of different origin. (A) Jurkat T cells were rendered apoptotic by irradiation with 150 Gy (Irrad), by incubation with staurosporine (Sts, 1 µM) or leucine zipper CD95 ligand (CD95L, 100 ng/ml) for 8 h. Externalization of PS, loss of membrane integrity and externalization of annexin A1 (AnxA1) was measured by flow cytometry using FITC-labeled annexin 5 and FITC-labeled DAC5 antibody. (B) The indicated cell types were rendered apoptotic by following treatments: activated primary human T cells and the cervix carcinoma cell line HeLa were incubated with staurosporine (1 µM), the hepatoma cell line HepG2 and the melanoma cell line A375 were irradiated with 150 Gy and 300 mJ/cm2 UV-C, respectively. Externalization of annexin A1 (AnxA1) was determined by flow cytometry using FITC-labeled DAC5 antibody (filled histograms), while membrane integrity was monitored by staining with 7-AAD. DAC5 staining on 7-AAD-negative cells is shown. The dashed line represents unstained cells. (C) Total human thymocytes were analyzed by flow cytometry for expression of CD4 and CD8. Staining with 7-AAD was used to exclude late apoptotic and necrotic cells. Externalized annexin A1 on 7-AAD negative cells was detected by FITC-labeled DAC5 antibody. Total thymocytes (left dot plot) and annexin A1-positive thymocytes (AnxA1+, right dot plot) are shown with respect to their CD4/CD8 expression. In the histograms on the left the percentages of PS-positive (PS) and annexin A1-positive (AnxA1) cells of total 7-AAD-negative thymocytes are indicated. Data are representative of at least 3 independent experiments. (TIFF) [file pone.0062449.s002.tiff]

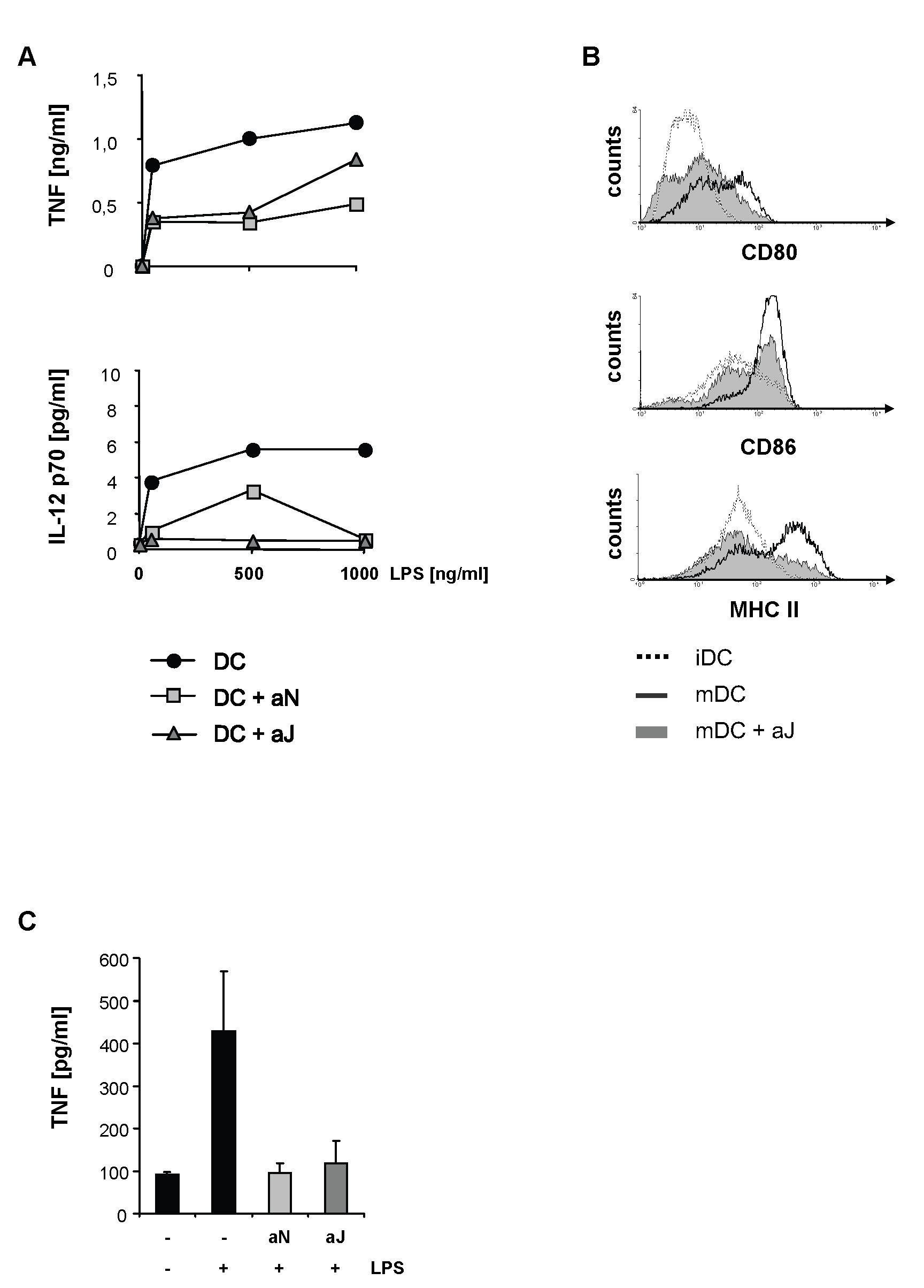

Supplement: Figure S3 — Apoptotic cells suppress TLR induced DC-activation. (A) Human DC were incubated with apoptotic neutrophils (aN) or apoptotic Jurkat T cells (aJ) for 4 h, or left untreated. After stimulation with the indicated concentrations of LPS for 12 hsecreted cytokines in culture supernatants were quantified by multiplex analysis. (B) For analysis of DC surface molecules, DC were pre-incubated with apoptotic Jurkat T cells as in (A) and subsequently stimulated by a cytokine cocktail for 2 days (mDC) or left untreated (iDC). iDC = untreated DC, dashed line; mDC = DC stimulated alone, bold line; mDC+aJ = DC stimulated after pre-incubation with apoptotic Jurkat T cells, filled histogram. (C) PMA-differentiated U937 cells were incubated with apoptotic neutrophils (aN) or apoptotic Jurkat T cells (aJ) for 4 h or left untreated, and subsequently stimulated with LPS (10 ng/ml) for 12 h. TNF concentrations in culture supernatants were determined by ELISA. Error bars represent means +/− SD of triplicate cultures. Data are representative of more than 3 independent experiments. (TIFF) [file pone.0062449.s003.tiff]

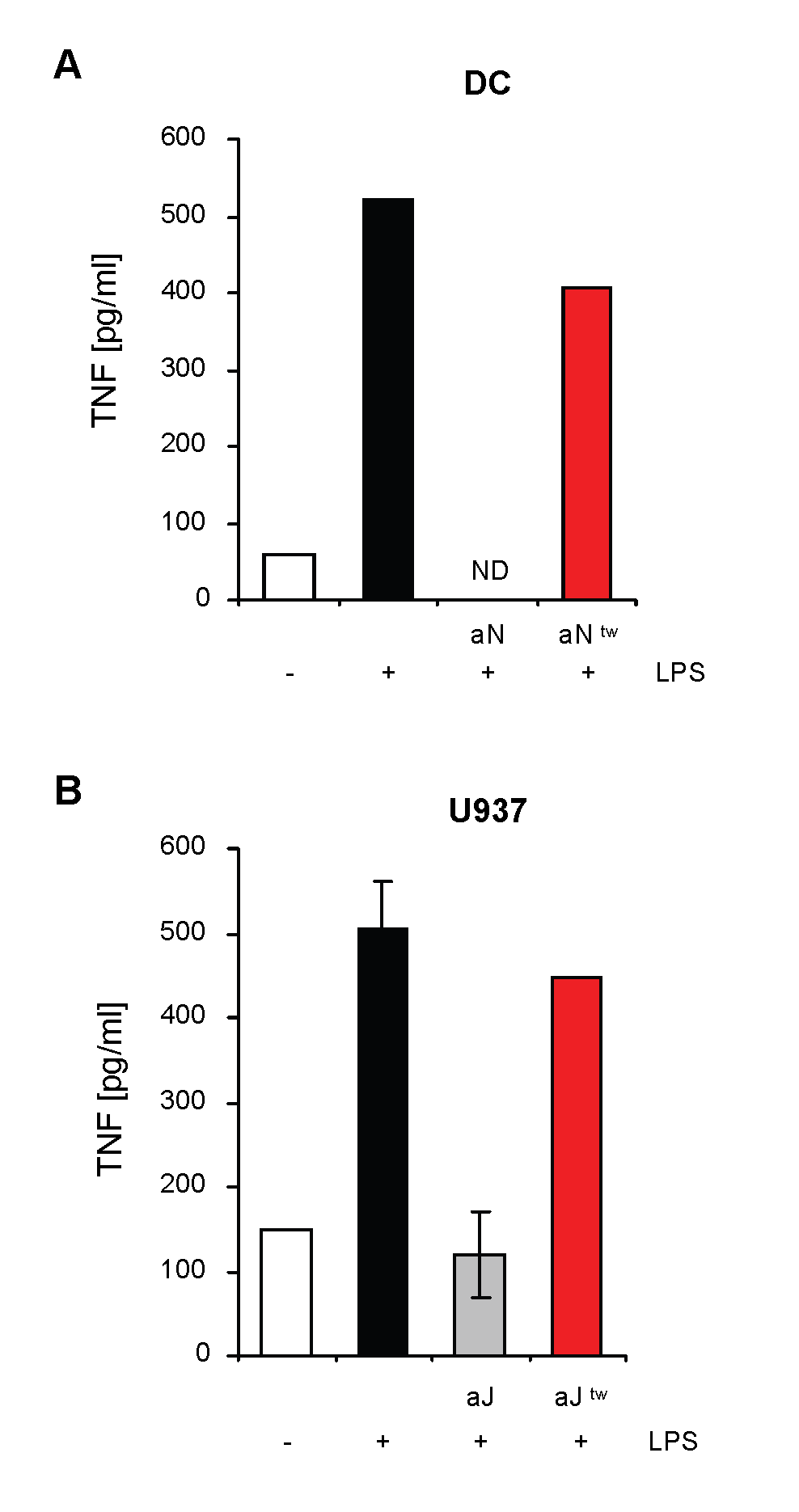

Supplement: Figure S4 — Suppression of DC by apoptotic cells is cell contact dependent. (A, B) Immature DC (A) or differentiated U937 cells (B) were incubated with apoptotic neutrophils (aN) or apoptotic Jurkat T cells (aJ) directly or in a transwell insert (aNtw; aJtw; 1 µm pore size) for 4 h. After stimulation with LPS (10 ng/ml) for 12–16 h, the concentration of TNF in culture supernatants was determined by ELISA. ND = not detectable. Error bars represent means +/− SD of duplicate wells. Data are representative of 3 independent experiments. (TIFF) [file pone.0062449.s004.tiff]

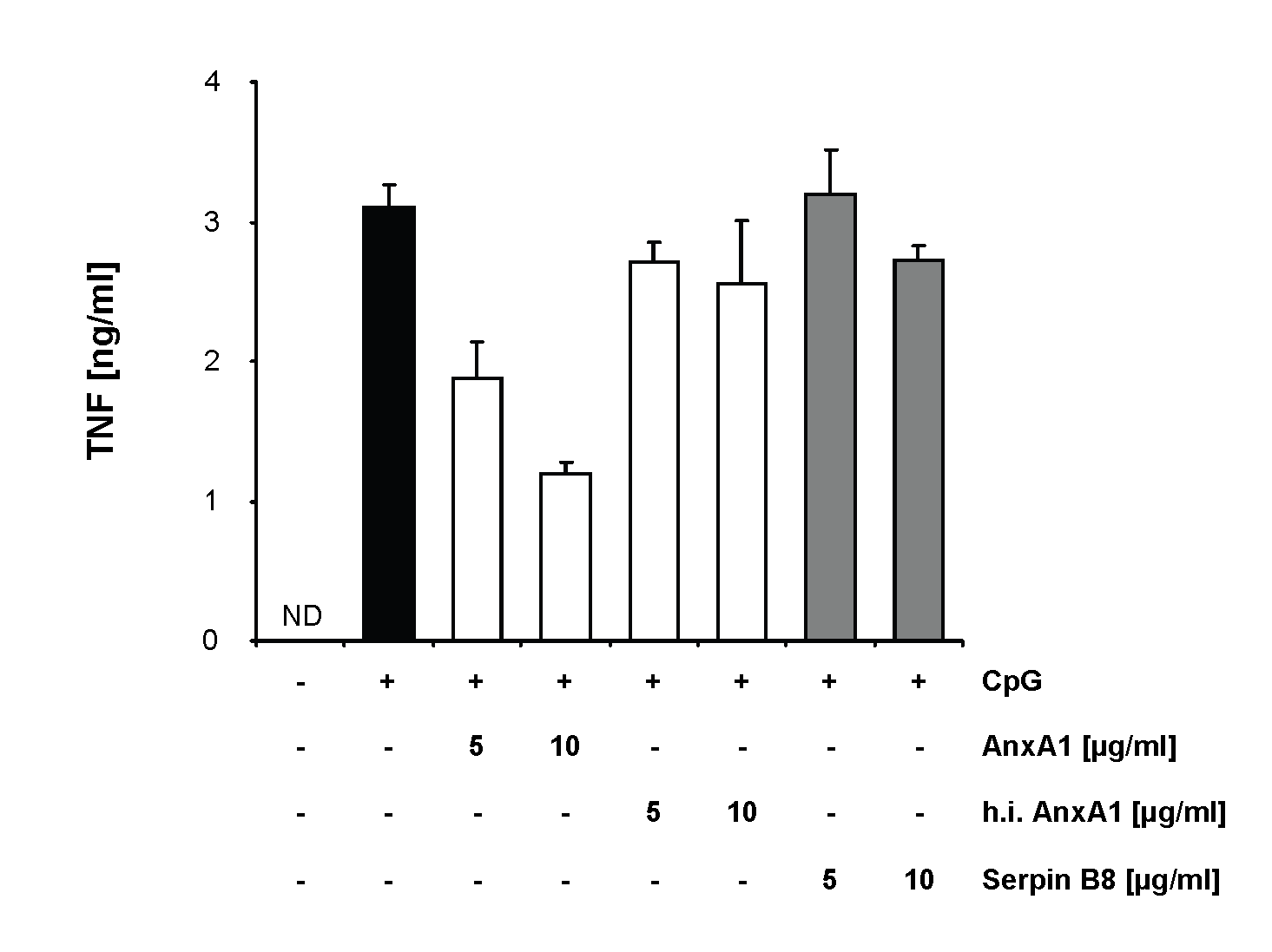

Supplement: Figure S5 — Annexin A1 suppresses TLR4−/− DC. Immature DC from TLR4−/− mice were incubated for 8 h with recombinant murine annexin A1 (AnxA1), heat inactivated annexin A1 (h.i. AnxA1), or recombinant serpin B8 as a control protein at the indicated concentrations. Subsequently, DC were stimulated with CpG (0.05 µM) for 12 h. TNF concentrations in culture supernatants were determined by ELISA. Error bars represent means +/− SD of duplicate cultures. Data are representative of more than 3 independent experiments. ND = not detectable. (TIFF) [file pone.0062449.s005.tiff]

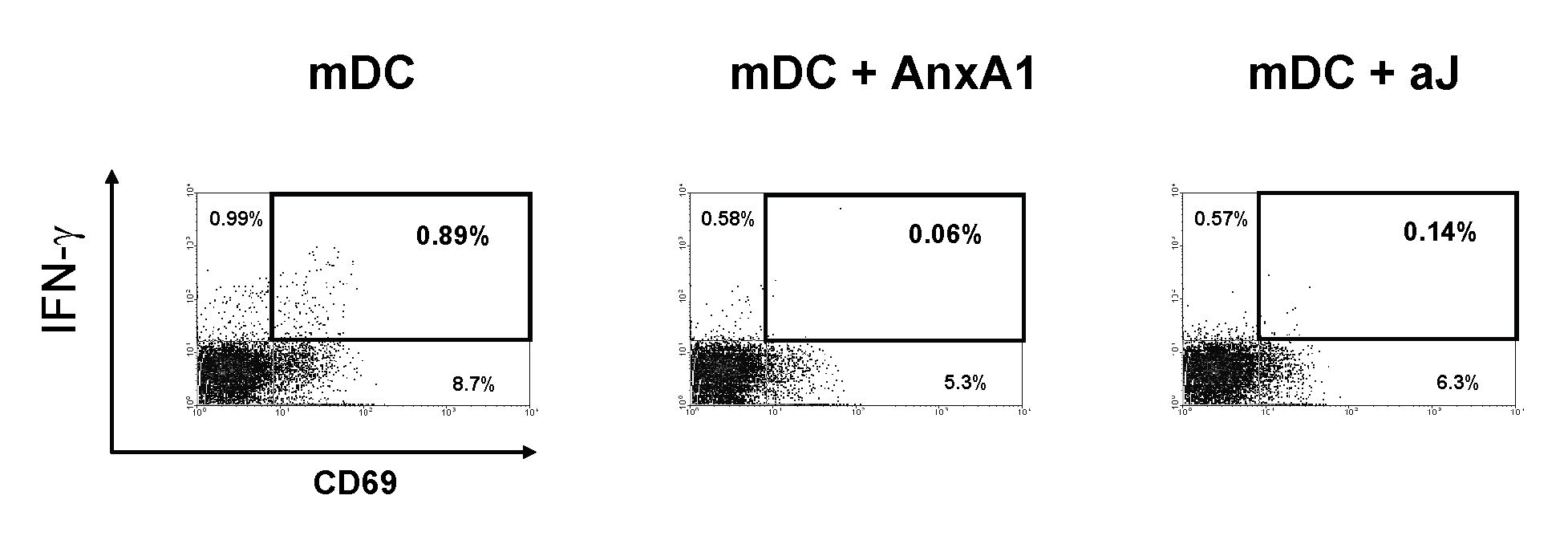

Supplement: Figure S6 — Intracellular T cell cytokines after stimulation by annexin A1-treated DC. Immature human DC were incubated overnight with annexin A1 (20 µg/ml, AnxA1), apoptotic Jurkat T cells (aJ), or left untreated. 2 days after maturation with R-848 (2.5 µg/ml), autologous CD4+ T cells together with SEB (0.1 ng/ml) were added to the culture wells. Intracellular T cell cytokines were analyzed by flow cytometry after 6 days of coculture with DC. Percentages of CD69+ and cytokine expressing T cells are indicated. Data are representative of 3 independent experiments. (TIFF) [file pone.0062449.s006.tiff]

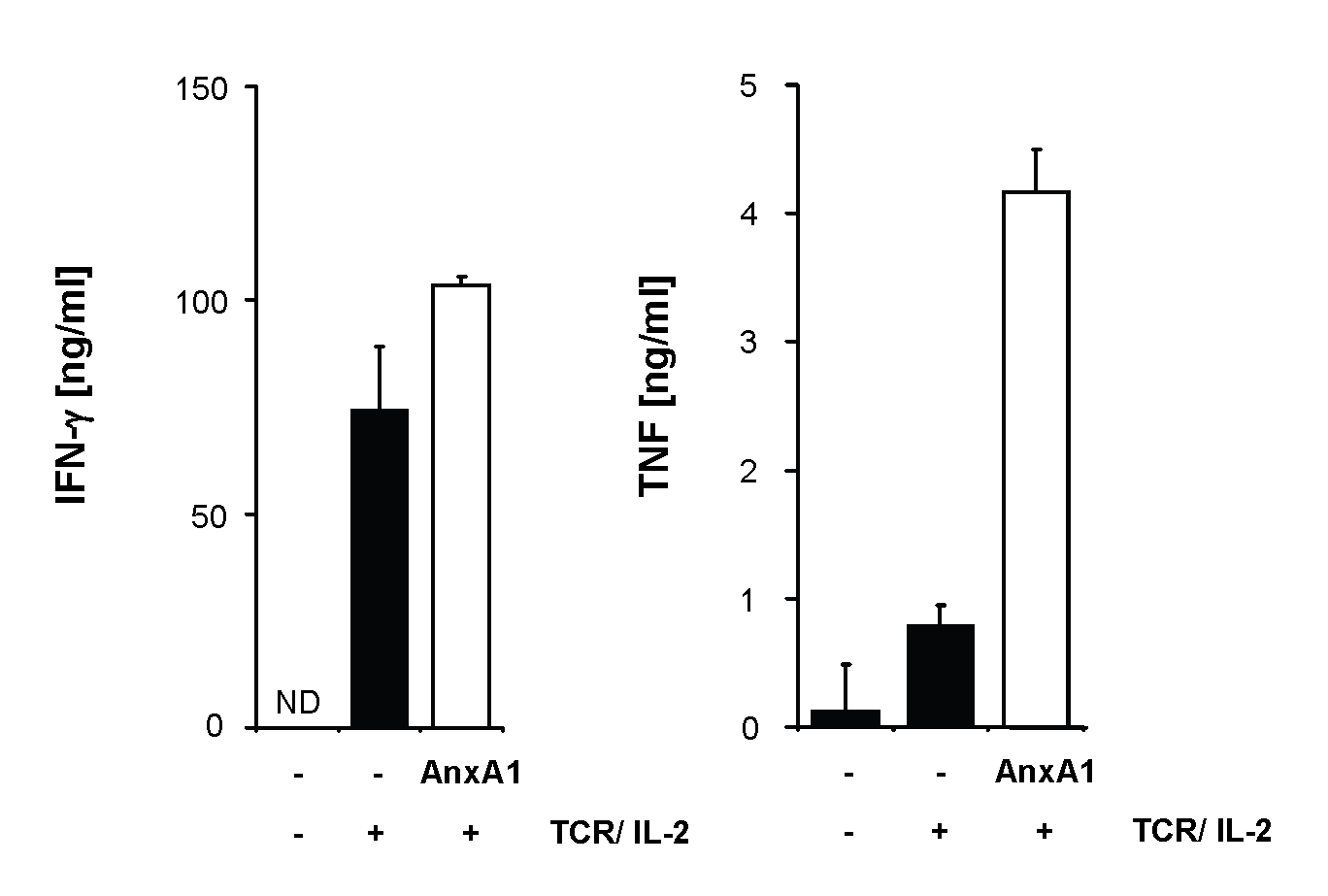

Supplement: Figure S7 — Enhanced Th1 cytokine secretion in annexin A1-treated T cells. CD4+ Tcells purified by magnetic beads (>95% purity) were incubated overnight with annexin A1 (20 µg/ml, AnxA1) followed by stimulation with agonistic antibodies against CD3 and CD28 together with IL-2 (TCR/IL-2; 1 µg/ml, 0.5 µg/ml and 25 U/ml, respectively) for 6 days. Subsequently, concentrations of cytokines in culture supernatants were determined by ELISA. Error bars represent means +/− SD of duplicate cultures. Data are representative of 3 independent experiments. (TIFF) [file pone.0062449.s007.tiff]

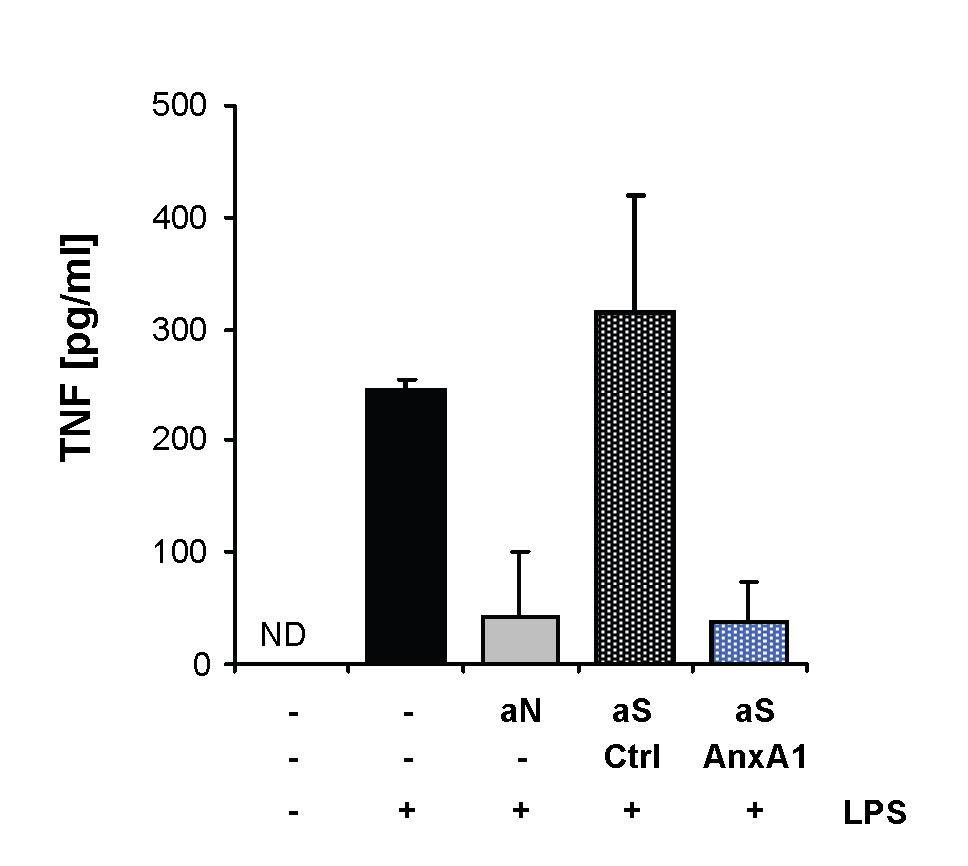

Supplement: Figure S8 — Apoptotic cells suppress activation of U937 cells via annexin A1. PMA-differentiated U937 cells were cocultured with apoptotic neutrophils (aN) or apoptotic Drosophila Schneider cells (aS) transfected with annexin A1 (AnxA1) or with a control plasmid (Ctrl). Subsequently, cultures were stimulated with LPS (5 ng/ml) for 12 h. TNF concentrations in culture supernatants were determined by ELISA. Error bars represent means +/− SD of duplicate cultures. Data are representative of 3 independent experiments. (TIFF) [file pone.0062449.s008.tiff]

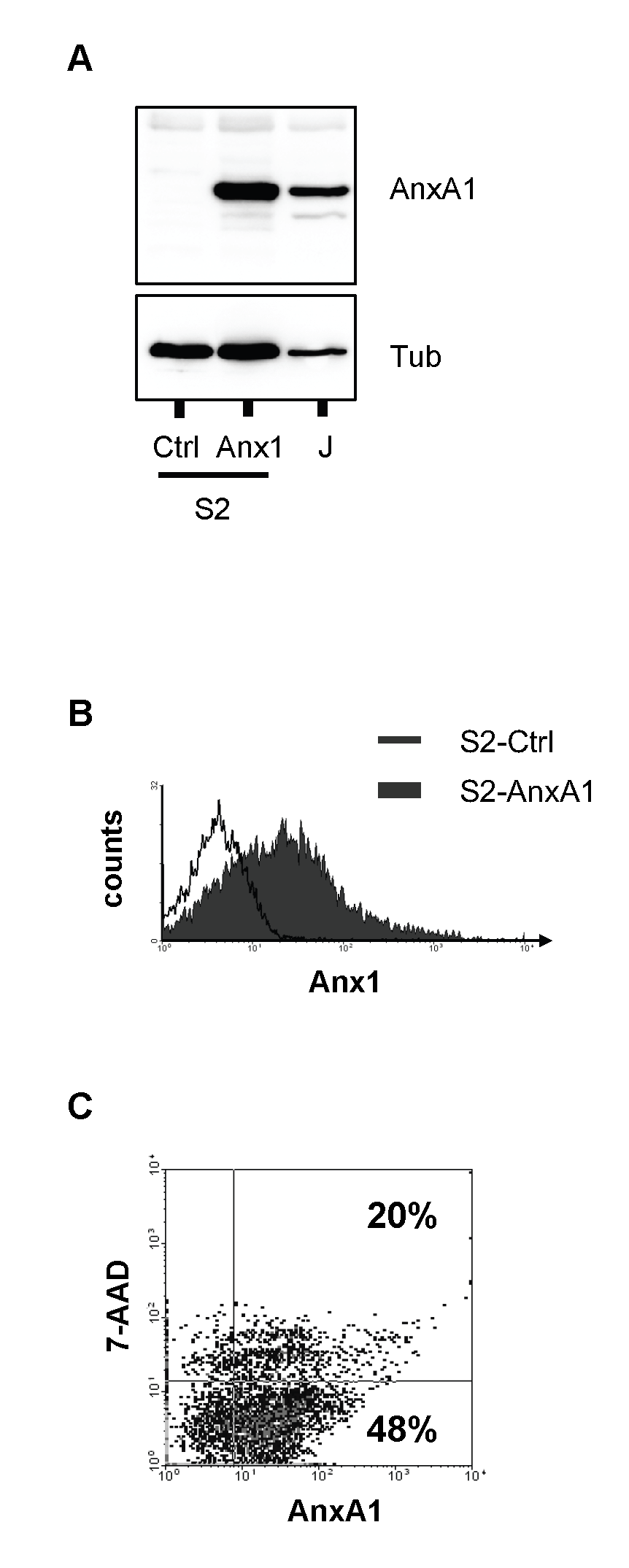

Supplement: Figure S9 — Transfected annexin A1 is externalized on apoptotic Drosophila Schneider cells. (A) Drosophila Schneider cells (S2) were transfected with human annexin A1 (AnxA1) or an empty control vector (Ctrl). Lysates of transfected Drosophila Schneider cells and Jurkat T cells (J) were analyzed for expression of annexin A1 (AnxA1) and tubulin (Tub). (B) 72 h after transfection, UV-C-irradiated (300 mJ/cm2, 12 h), apoptotic Drosophila Schneider cells transfected with annexin A1 (S2-AnxA1, filled histogram) or a control plasmid (S2-Ctrl, bold line) were stained with FITC-labeled DAC5 antibody. (C) Apoptotic Drosophila Schneider cells transfected with annexin A1 as in (B) were tested for annexin A1 externalization using FITC-labeled DAC5 antibody, while membrane integrity was monitored by staining with 7-AAD. Percentages indicate annexin A1 (AnxA1)-positive cells subdivided into early apoptotic cells with intact cell membrane (7-AAD-negative) and late apoptotic cells (7-AAD positive). Data are representative of more than 3 independent experiments. (TIFF) [file pone.0062449.s009.tiff]

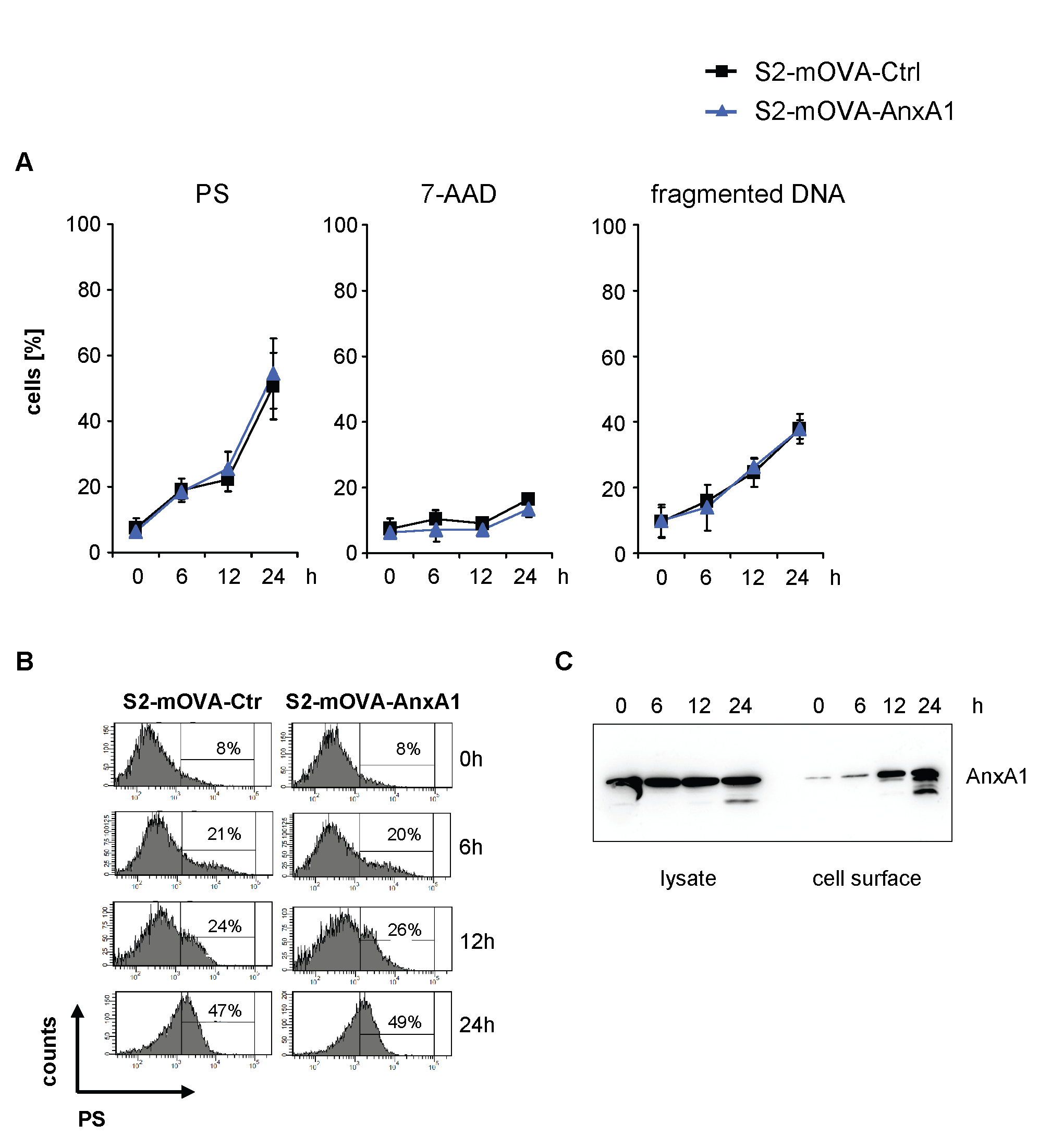

Supplement: Figure S10 — Kinetics of S2 cell apoptosis. (A–C) Drosophila S2 cells expressing membrane-anchored ovalbumin and murine annexin A1 (S2-mOVA-AnxA1) or a control plasmid (S2-mOVA-Ctrl) were irradiatied with UV-C light (300 mJ) and analyzed after indicated time periods by flow cytometry. Kinetics (A) and representative histograms of PS-exposure (B) are shown. Externalization of PS and loss of membrane integrity were assessed by staining with FITC-labeled annexin A5 and 7-Amino-actinomycin D (7-AAD), respectively. Nuclei with fragmented DNA were detected as described by Nicoletti et al. In parallel, aliquots of cells were resuspended in PBS/EDTA (5 mM). Murine annexin A1 (AnxA1) in lysates and EDTA-washes (cell surface) of S2-mOVA-AnxA1 cells was detected on Western blot using a rat monoclonal antibody generated in our lab (C). Error bars represent means +/− SD of 3 experiments. (TIFF) [file pone.0062449.s010.tiff]

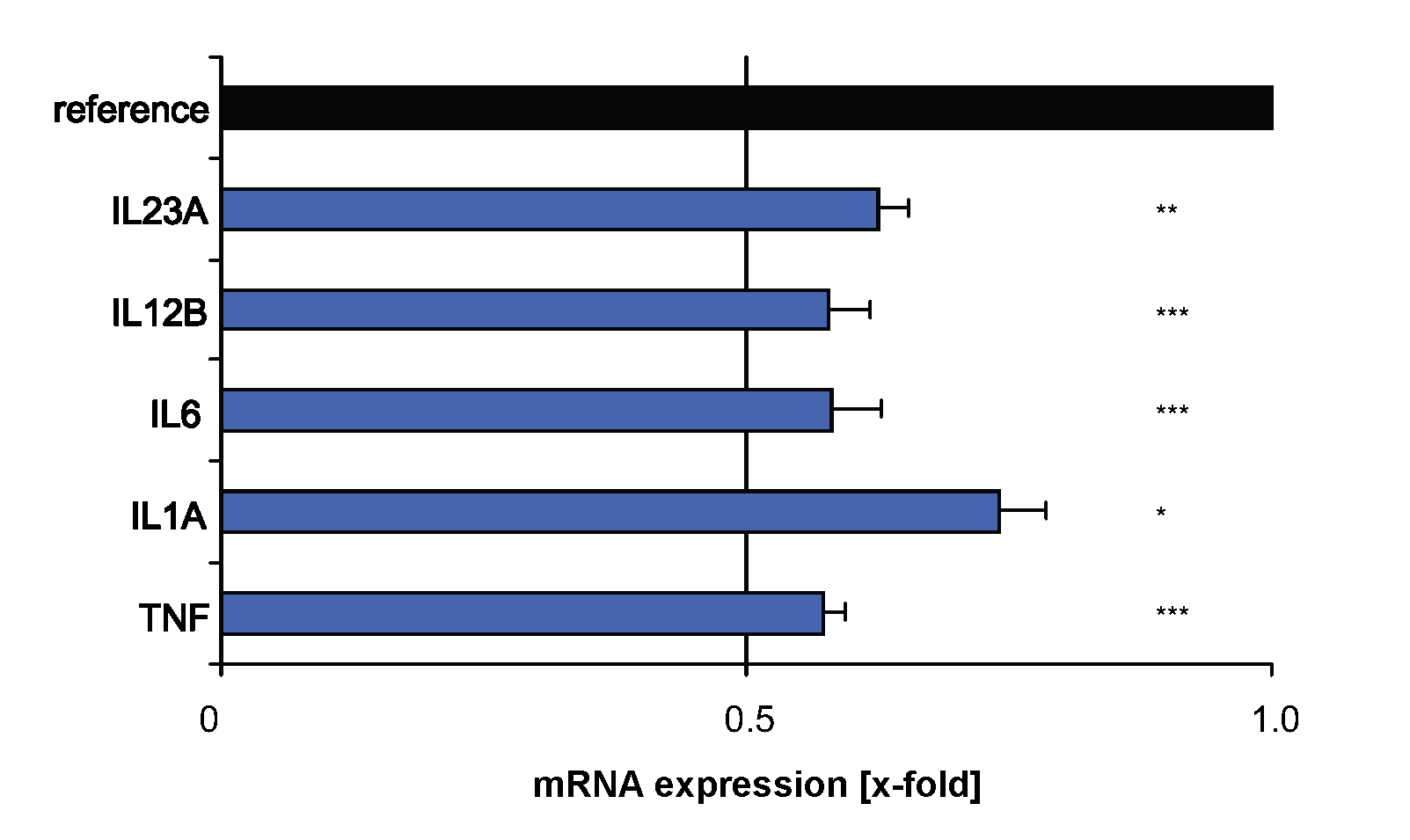

Supplement: Figure S11 — Annexin A1 suppresses gene transcription of NF-κB-dependent cytokines. Human DC were incubated with annexin A1 (10 µg/ml) or left untreated. Subsequently, DC were stimulated with R-848 (1 µg/ml) for 2–3 h, and mRNA expression was analyzed by reverse transcription quantitative PCR. Shown are mRNA expression data of annexin A1-treated, TLR-stimulated samples relative to TLR-stimulated samples only (reference). Error bars represent means +/− s.e.m. *P<0.05; **P<0.001 (n = 5–12 donors). (TIFF) [file pone.0062449.s011.tiff]

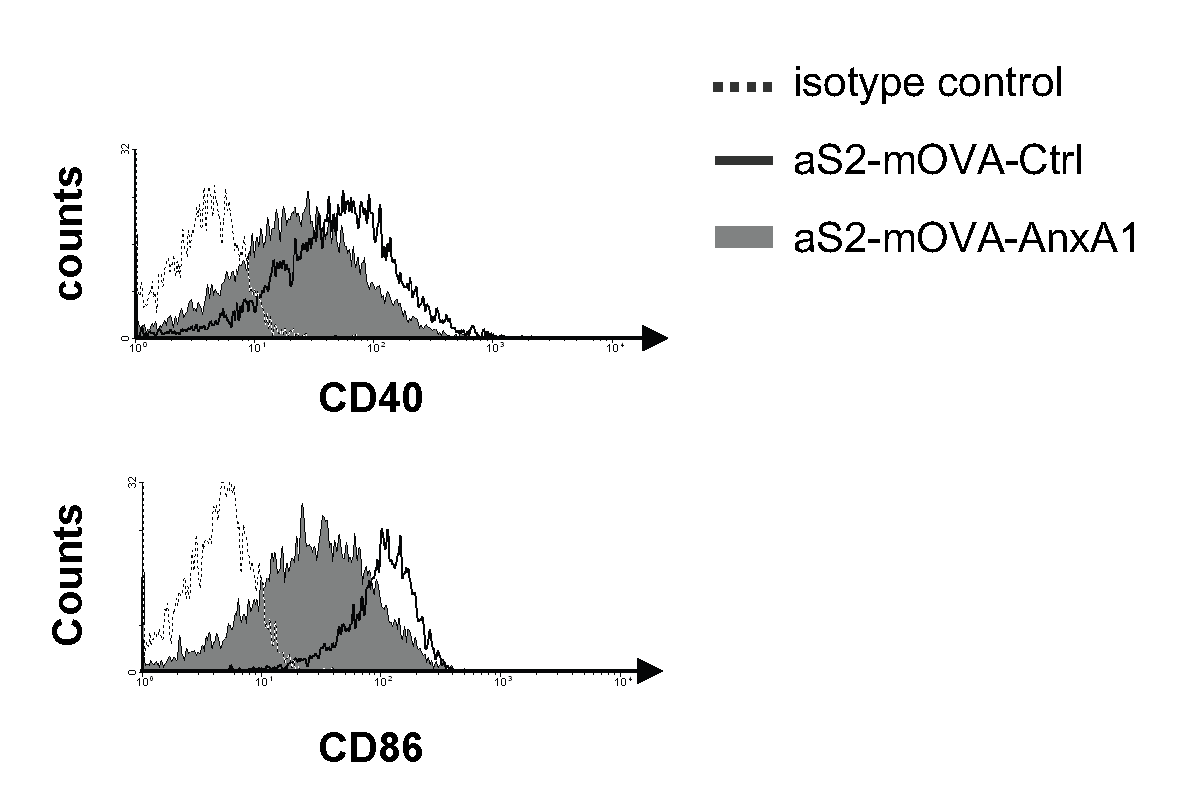

Supplement: Figure S12 — Annexin A1 inhibits DC activation in vivo . Apoptotic, mOVA-expressing Drosophila Schneider cells (aS2-mOVA) transfected with annexin A1 (AnxA1) or a control plasmid (Ctrl) were injected into mice. After 2 days, DC in lymph nodes of S2-injected mice were analyzed for CD40 and CD86 expression by flow cytometry. Shown are representative histograms of lymph node cells of mice injected with the indicated apoptotic S2 cells and gated on CD11c+ and MHC class II+ cells. Data are representative of 3 independent experiments. (TIFF) [file pone.0062449.s012.tiff]

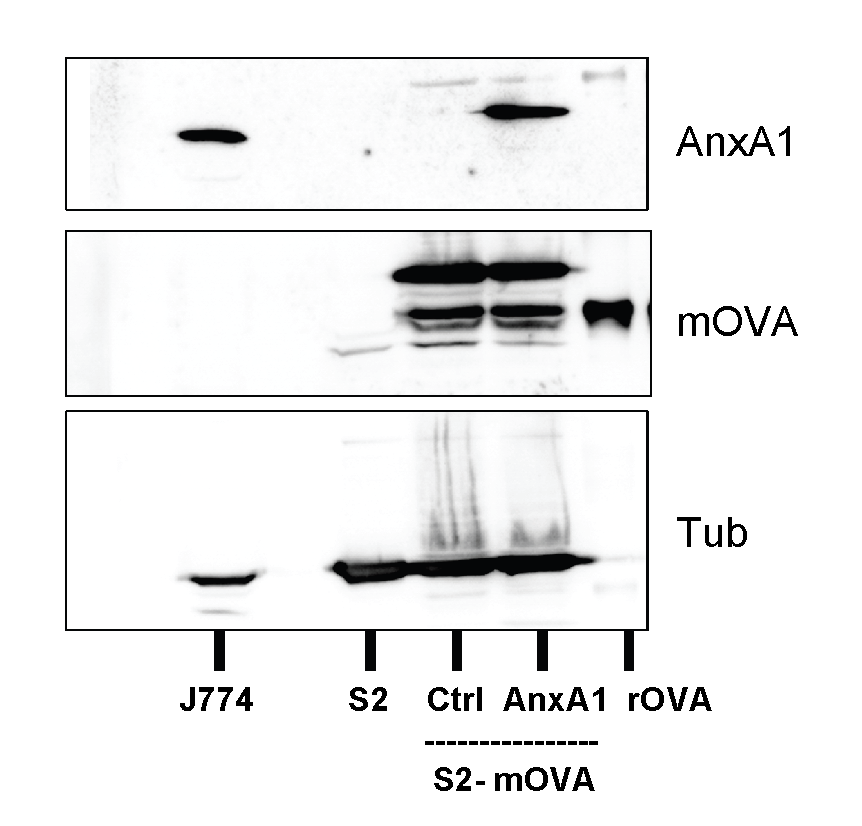

Supplement: Figure S13 — Annexin A1 and mOVA expression in S2 cells injected into mice. Lysates of S2 cells (5 million cells/lane) stably transfected with mOVA (S2-mOVA) and murine annexin A1 (AnxA1) or a control vector (Ctrl) were analyzed on Westernblot using antibodies against OVA, murine annexin A1 and tubulin (Tub), respectively. For comparison, lysates of untransfected S2 cells (S2), of the murine macrophage cell line J774 (1 million cells/lane) and recombinant OVA (rOVA, 2.5 ng/lane) were loaded in parallel. (TIFF) [file pone.0062449.s013.tiff]
